# Oral and Topical Anti-Inflammatory Activity of *Jatropha integerrima* Leaves Extract in Relation to Its Metabolite Profile

**DOI:** 10.3390/plants11020218

**Published:** 2022-01-14

**Authors:** Engy A. Mahrous, Ahmed H. Elosaily, Abeer A. A. Salama, Ahmed M. Salama, Soheir M. El-Zalabani

**Affiliations:** 1 Department of Pharmacognosy, Faculty of Pharmacy, Cairo University, Cairo 11562, Egypt; soheir.elzalabani@pharma.cu.edu.eg; 2 Department of Pharmacognosy, Faculty of Pharmacy, Ahram Canadian University, Giza 12573, Egypt; elosaily1987@hotmail.com (A.H.E.); am.salama@hotmail.com (A.M.S.); 3 Pharmacology Department, National Research Center, Cairo 12622, Egypt; berrotec@yahoo.com

**Keywords:** anti-inflammatory, diterpenoids, cyclic peptide, Euphorbiaceae, hydroxyl fatty acids *Jatropha integerrima*, vitexin, UPLC/MS-MS

## Abstract

*Jatropha integerrima* Jacq., family: Euphorbiaceae, is used in India and subtropical Africa to treat different skin conditions. In this study we evaluated the anti-inflammatory activity of *J. integerrima* leaves extract (JILE) using rat paw edema model. The extract was administered orally (200 and 400 mg/kg) or applied topically as creams at 2.5, 5, and 10% strength. Four hours post-treatment, maximum reduction of edema volume by 63.09% was observed after oral administration of JILE (400 mg/kg) as compared to indomethacin with 60.43%. The extract anti-inflammatory effect was accompanied by a decrease in NO, prostaglandin PGE2, TNF-α and PKC levels by 19, 29.35, 16.9, and 47.83%, respectively. Additionally, topical applications of JILE showed dose dependent reduction in paw edema and resulted in normalized levels of PGE2, TNF-α, and PKC when used as 10% cream. Signs of inflammations were reduced or absent from paw tissue of animals receiving JILE either orally or topically. Finally, liquid chromatography/mass spectrometry analysis of JILE resulted in the annotation of 133 metabolites including 24 diterpenoids, 19 flavonoids, 10 phenolic acid conjugates, 8 cyclic peptides, 6 phytosterols, 4 sesquiterpenes, and 4 coumarins. Several of the annotated metabolites have known anti-inflammatory activity including vitexin, isovitexin, fraxitin, scopeltin, stigmasterol, and many diterpenoidal derivatives.

## 1. Introduction

The genus *Jatropha* (family Euphorbiaceae) has a wide distribution in tropical and subtropical regions especially South America, West Africa, India, and Southeast Asia [1,2]. Many members of the genus were used medicinally in their indigenous countries such as *J. gossypifolia*, *J. curcas*, *J. chevalieri,* and *J. multifida* [3]. Leaves and latex from *Jatropha* plants are especially useful in treating skin conditions such as ulcers, blisters, eczema and also to accelerate wound healing [4,5,6]. The genus is rich in bioactive secondary metabolites especially diterpenoids of tigliane, lathyrane, and jatrophane skeletons which exist mainly as esters [7,8]. However, among more than 175 *Jatropha* species, only few species were chemically investigated. As a result of these investigations, many structurally unique and bioactive phytochemicals were identified including flavonoids, cyclic peptides, lignans, and diterpenes [1,3].

*Jatropha integerrima* Jacq (*syn. Jatropha pandurifolia* And.), also known as spicy jatropha, is cultivated around the world as an ornamental shrub due to its showy bright red flowers. Its leaves are used as poultice in India and Bangladesh to treat different conditions including eczema, pruritus and skin warts [9,10]. Phytochemical investigation of *J. integerrima* identified coumarins [11], cyclic peptides [12], neolignans [13], and several novel diterpenes with different biological activities [14,15]. More than 16 new compounds were isolated from *J. integerrima* in the past decade alone [13,14,15], indicating a rich metabolome that is yet to be fully explored. Recent pharmacological investigation of the plant showed promising antimicrobial activity of extracts and essential oils obtained from seeds and leaves [16,17] and strong antioxidant activity of the flowers extract [18].

Based on the accumulated literature and the known folk use of *J. integerrima*, this study was performed to investigate the anti-inflammatory effect of *J. integerrima* leaves extract (JILE) when administered orally or applied topically. Anti-inflammatory activity was assessed in rat paw edema model through measuring edema volumes and levels of inflammatory mediators nitric oxide (NO), tumor necrosis factor-α (TNF-α), prostaglandin E2 (PGE2), and protein kinase C (PKC) in addition to examining histopathological features of paw tissue. Furthermore, ultra-performance liquid chromatography coupled to high resolution mass spectrometry (UPLC-MS) was used to identify secondary metabolites in JILE through tandem mass fragmentation to assist with identifying metabolites that can contribute to the anti-inflammatory activity of the plant.

## 2. Results

### 2.1. Acute Toxicity Study

Rats treated with *J. integerrima* leaves extract (JILE) at a high dose of 5 g/kg did not show any skin abnormalities or changes in respiratory, circulatory, and somatomotor activities as well as behavior pattern.

### 2.2. Evaluation of Oral Anti-Inflammatory Effect of Jatropha integerrima Leaves Extract

**Figure 1:**
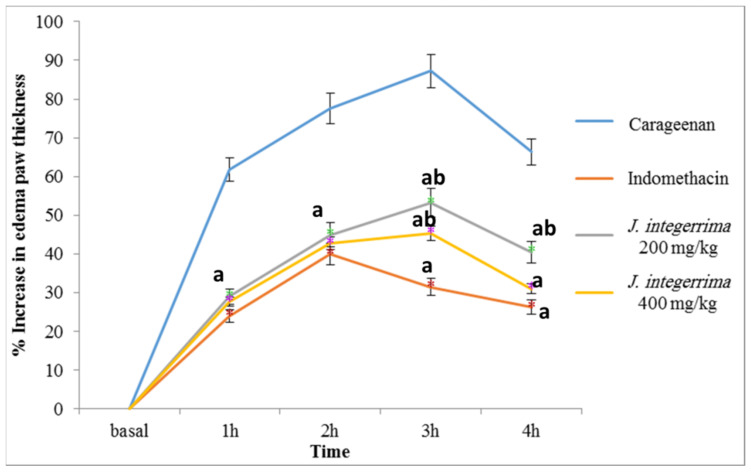
Effect of oral administration of ethanol extract of *Jatropha integerrima* leaves on edema volume. One way ANOVA and Fishers LSD comparison test were used, *p* < 0.05; a: significantly different from carrageenan control group at respective time point; b: significantly different from indomethacin group at respective time point.

The subplanter injection of 100 μL of 1% sterile carrageenan into the rat hind paw elicited an inflammation manifested by swelling, erythema and a time-dependent increase in paw edema by 61.82, 77.58, 87.24%, and 66.37% after 1, 2, 3, and 4 h after injection, as compared with precarrageenan control values, Figure 1. This observed response to carrageenan injection follows the same pattern of maximum inflammation after 3 h as previously reported [19].

Pretreatment with JILE (200 mg/kg and 400 mg/kg, orally) showed a significant inhibition of edema formation by 53.09% and 55.17% after 1 h, 42.15% and 44.87%, after 2 h, 39.04% and 48.03% after 3 h, and 39.01% and 63.90% after 4 h, respectively, as compared with carrageenan control group at the same time. In the same assay, indomethacin treatment (25 mg/kg) resulted in a 61.17%, 48.56%, 63.9%, and 60.43% reduction of edema volume, as compared with that of carrageenan group after 1, 2, 3, and 4 h postcarrageenan injection, respectively, Figure 1.

By the end of the experiment, serum NO level increased by 44% in animals that received only carrageenan as compared with normal control group. Animals that were pre-treated with indomethacin and *J. integerrima* at 200 mg/kg and 400 mg/kg had significantly decreased serum NO levels by 17%, 13%, and 19%, respectively, as compared with that of animals that received carrageenan only (*p* < 0.05), Table 1.

Similarly, after 4 h following carrageenan injection, serum levels of inflammation mediators such as PGE2, TNF-α, and PKC were elevated by 98.5%, 38.75%, and 72.94%, respectively, as compared with that of normal control group. Animals pretreated with indomethacin had their levels of PGE2 decreased by 28.85%, TNF-α by 17.76%, and PKC by 44%. Meanwhile, pretreatment with JILE at 200 mg/kg caused insignificant changes in serum levels of TNF-α and PGE2 (*p* > 0.05) but reduced PKC by 46.47%, Table 1. Meanwhile, pretreatment with JILE at a dose of 400 mg/kg decreased PGE2 by 29.35%, TNF-α by 16.90% and PKC by 47.83%, as compared with carrageenan control group resulting in restoring serum levels of NO and PKC to their basal levels and were not significantly different from the effect observed with indomethacin treatment (*p* > 0.05).

**Figure 2:**
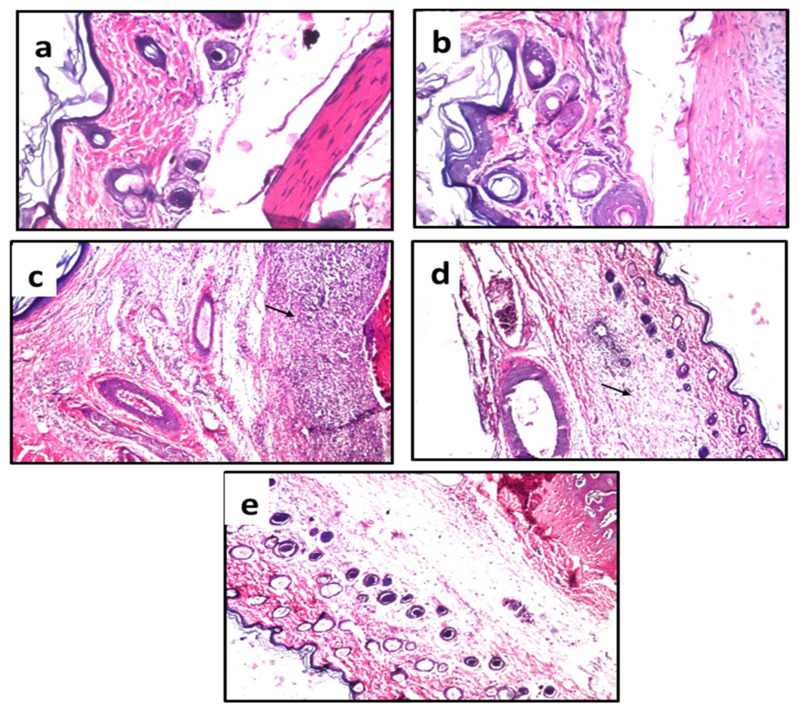
Photomicrographs of subcutaneous and dermal tissues of treated rats. (**a**) control group; (**b**) indomethacin group; (**c**) carrageenan group; (**d**) group receiving oral 200 mg/kg of *J. integerrima* leaves extract; and (**e**) group receiving oral 400 mg/kg of *J. integerrima* leaves extract. Arrows refer to inflammatory cell infiltration.

The paw tissue in the healthy group and the group treated with carrageenan and indomethacin displayed normal histological features (Figure 2a,b), while the carrageenan treated group showed a few inflammatory cells infiltration in the dermal tissue and massive inflammatory cell infiltration in the subcutaneous tissue (Figure 2c). Mild focal inflammatory aggregation in subcutaneous tissue was noticed in the group treated with JILE (200 mg/kg) with normal dermal tissue (Figure 2d) while the dermal and subcutaneous tissues appeared intact in the group treated with JILE at 400 mg/kg, (Figure 2e).

### 2.3. Evaluation of Topical Anti-Inflammatory Activity of Jatropha integerrima Leaves Extract

**Figure 3:**
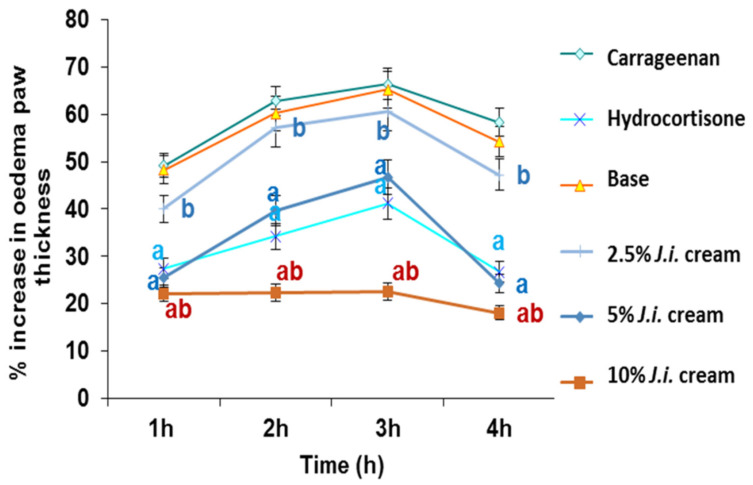
Effect of topical administration of *Jatropha integerrima* cream on edema volume in rat hind paw model. One way ANOVA and Fishers LSD comparison test* were used in data analysis, *p* < 0.05; a significantly different from carrageenan control value at respective time point. b significantly different from hydrocortisone group value at respective time point.

Rats given *J. integerrima* cream in the skin irritation test showed no sensitivity or irritation. The subplantar injection of carrageenan into the rat hind paw elicited an inflammation as previously mentioned in Section 2.2. Topical application of base non-medicated cream and 2.5% strength JILE cream before carrageenan injection showed no significant inhibition of edema formation at all-time points as compared with that of carrageenan group, Figure 3. Meanwhile topical application of higher strength JILE creams (5% and 10%) showed a significant inhibition of edema formation at all-time points especially after 4 h by 58.27% and 68.96%, respectively, as compared with that of untreated animals. In addition, the effect produced upon application of 10% JILE cream was superior to standard hydrocortisone cream (*p* < 0.05), Figure 3.

Four hours postcarrageenan injection, inflammatory mediators were measured. An increased serum NO levels by 39.66% and 35.81% were observed in carrageenan treated animals and in animals treated with base cream, respectively, as compared with that of healthy control group. Animals that were treated with hydrocortisone cream and JILE cream at 2.5%, 5% and 10% showed significantly decreased serum NO levels by 14.84%, 10.92%, 17.04%, and 18.04%, respectively, as compared with that of untreated animals, Table 2.

As expected for animals receiving carrageenan only or treated with base cream, levels of inflammatory mediators were all elevated. An increase of serum levels by 32.86% and 32.03% in case of PGE2, 21.89% and 18.95% for TNF-α, and 47.37% and 46.43% in case of PKC were observed in animals that received no treatment or base cream only, respectively. Treatment with hydrocortisone resulted in a decrease in serum levels of PGE2 by 21.94%, TNF-α by 15.12%, and PKC by 32.13% when compared with that of untreated group. Meanwhile, pretreatment with cream containing 2.5% of JILE decreased PGE2 and PKC levels by 10.41% and 23.32%, respectively, while animals treated with cream containing 5% JILE decreased PGE2, TNF-α and PKC by 14.95%, 6.51%, and 26.58%, respectively, as compared with that of carrageenan group. Meanwhile, animals that received cream containing 10% of extract showed normalized serum levels of TNF-α, PKC, and PGE2, as compared with that of control group, as did treatment with hydrocortisone cream, Table 2.

**Figure 4:**
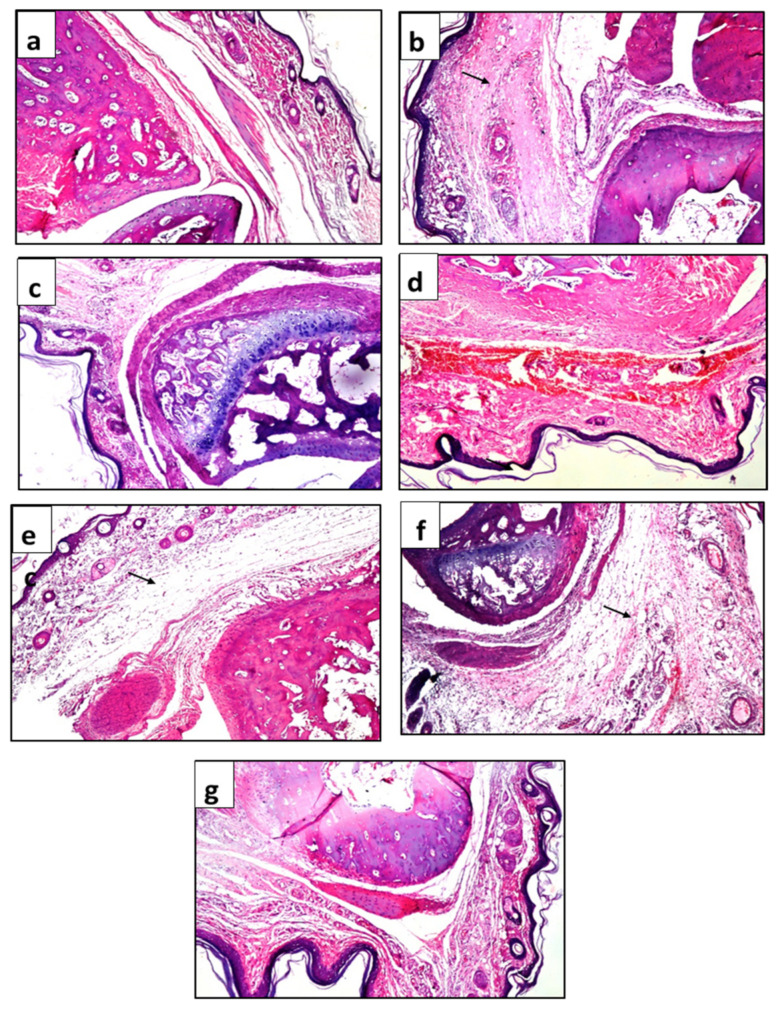
Photomicrographs of subcutaneous and dermal tissues of treated rats. (**a**) healthy animals; (**b**) animals treated with carrageenan only; (**c**) 1% hydrocortisone cream; (**d**) base cream; (**e**) 2.5% JILE cream; (**f**) 5% JILE cream; (**g**) 10% JILE cream. Arrows refer to inflammatory cell infiltration.

The paw tissue in the healthy group and group treated with carrageenan and hydrocortisone displayed normal histological features (Figure 4a,c), except for few inflammatory cells in the hydrocortisone group. Animals that were injected with carrageenan but received no further treatment showed massive inflammatory cell infiltration in the subcutaneous tissue (Figure 4b). Hemorrhages, inflammatory cells, and hyalinization were noticed in animals treated with base cream only (Figure 4d). Edema with inflammatory cells infiltration was detected in animals treated with creams containing 2.5% and 5% of JILE (Figure 4e,f), while animals treated with 10% JILE cream showed few inflammatory cells in subcutaneous tissue, and the skin layer remained intact, Figure 4g.

### 2.4. UPLC-MS Analysis of Jatropha integerrima Extract

**Figure 5:**
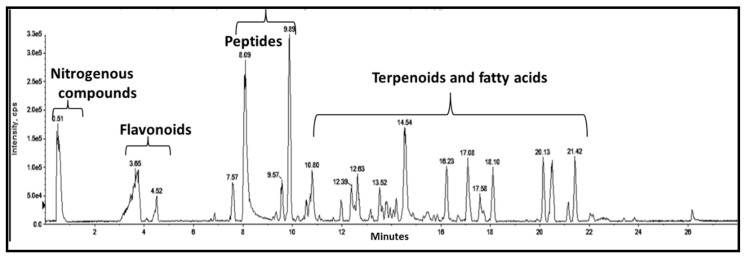
Base peak chromatogram of the UPLC/MS analysis of *J. integerrima* leaves extract.

A total of 133 compounds were annotated in the extract representing different primary and secondary metabolites. Flavonoids and amino acids were eluted between 0–6 min, cyclic peptides were eluted from 9–11 min while diterpenoids, sterols, fatty acids, and fatty acid glycerides were eluted at the late part of the chromatographic run, Figure 5.

#### 2.4.1. Phenolic Compounds

**Figure 6:**
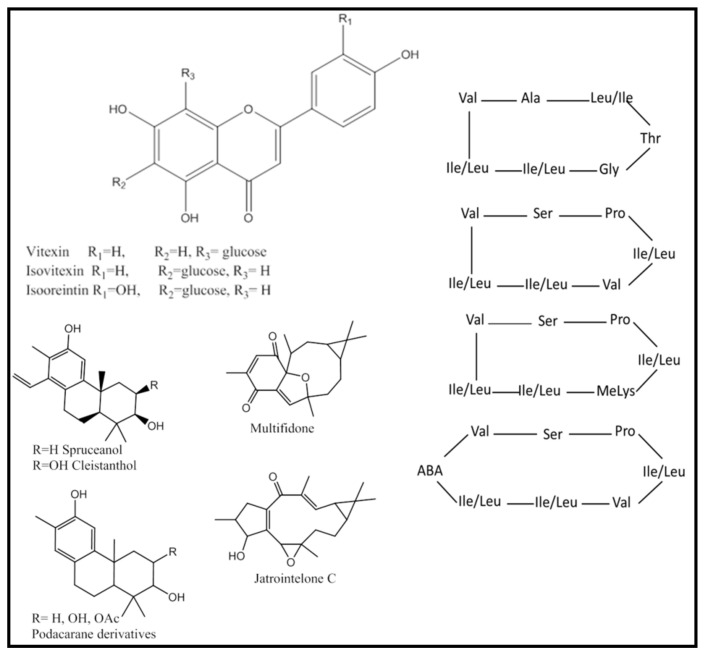
Structures of selected compounds annotated in *J. integerrima* extract including flavonoids, peptides, and diterpenoids.

Analysis of the UPLC/MS chromatogram resulted in the identification of 19 flavonoids, 10 phenolic acid conjugates, 4 coumarins, and 1 lignan. Flavonoids occurred mainly as *C*-linked hexoses (11 flavonoids) which were readily identified by the neutral loss of 120 Da, Figure S1, as observed in peaks 10, 11, 13, 14, 19, 22, 24, 29, 30, 32 and 35 (Table 3, Figure 6). Most of the *C*-glycosides were derivatives of apigenin, including isoorientin (peak 11), vitexin (peak 13), and isovitexin (peak 14), which are reported here for the first time from *J. integerrima*. Vitexin (C-8 glycoside) was differentiated from its structural isomer isovitexin (C-6) by the intensity of the fragment ions at *m*/*z* 313 and 283, produced due to the cleavage of the C-attached sugar, Figure S2.

Two *C*-glycosides dimers were observed at 5.25 and 5.94 min (peaks of 30 and 32, respectively) with [M + H]^+^ at *m*/*z* 877.218 for C_30_H_41_O_20_ and were assigned to the isomeric jatrophenols I/II/III previously isolated from *J. multifida* [20]; they were also detected in two other *Jatropha* species [21]. These are dimers of isovitexin connected by a methylene bridge [22], Figure S3. Fragmentation of these dimers resulted in loss of isovitexin monomer (−432 Da) and appearance of methylisovitexin fragment ion at *m*/*z* = 445, Table 3, Figure S3. Two other peaks (peaks 19 and 35) produced the same fragment ion (*m*/*z* = 445) with [M+H]^+^ observed at *m*/*z* 560.1788 (for C_27_H_30_NO_12_) and 736.2208 (for C_33_H_37_NO_15_), respectively. Both peaks showed loss of proline amino acid as indicated by the neutral loss of 115 Da for C_5_H_9_NO_2_, Figure S4 and S5 and produced methylisovitexin fragment ions at *m*/*z* = 445 and 427. Therefore, peak 19 and 35 were annotated as methylisovitexin proline and methylisovitexin proline ferulate. To the best of our knowledge, this is the first report of isovitexin proline derivatives.

Meanwhile, flavonoids *O*-glycosides were characterized by the neutral loss of 162 Da for hexoses (peaks 10, 16, 22, 26), Figure S6, and 146 for rhamnosyl residue (peak 25) [23], Figure S7, Table 3.

Phenolic acids in *J. integerrima* extract were detected only as conjugated molecules substituted with medium or long aliphatic chains, as seen in peaks 52, 56, 74, 76,106, 118, 128, and 129. Among the annotated phenolic acid esters, only tetradecyl ferulate (peak 118) was previously reported in the genus and was isolated from both *J. curcas* [24] and *J. multifida* [25].

#### 2.4.2. Nitrogenous Compounds

Eight simple nitrogenous compounds were annotated in *J. integerrima* extract, including choline and its glycoside (peaks 4 and 1, respectively). Other simple nitrogenous compounds included proline (peak 5) and its betaine derivative stachydrine (peak 6) in addition to proylproline (peak 8).

Apart from the simple nitrogenous compounds, 8 cyclic peptides (hepta-and octa-peptides) were observed between retention time of 9.5–10.5 min. Five cyclic peptides were previously isolated from the latex of *J. integerrima* [12,26,27] including integerrimide A (peak 53, [M + H]^+^ at *m*/*z* 781.4595 calculated for C_40_H_61_N_8_O_8_) [12], integrimmacyclopeptide A (peak 54, [M + H]^+^ at *m*/*z* 767.5031 calculated for C_37_H_67_N_8_O_9_), and integrrimacyclopeptide B (peak 47, [M + H]^+^ at *m*/*z* 652.4030 calculated for C_31_H_54_N_7_O_8_). Additionally, four cyclic peptides, which were not previously reported, were annotated in peaks 43, 45, 46, and 55, Figure 6. The amino acid sequence of these peptides was tentatively determined based on the high-resolution mass of their fragment ions Table S1, Figures S8–S11. These new cyclic peptides consisted mainly of hydrophobic residues including leucine or isoleucine (neutral loss of 113 Da, corresponding to C_6_H_11_NO) and valine (neutral loss of −99 Da corresponding to C_5_H_9_NO), Table S1, Figure 6.

#### 2.4.3. Terpenoids

The most abundant group of secondary metabolites detected in *J. integerrima* leaves was that of terpenoids including sesquiterpenes (4 metabolites), diterpenes (24 metabolites) and two triterpenoids. To date, only free diterpenoids or their monoacetyl derivatives were reported in *J. integerrima* [14,15,28]. In this study, free diterpenoids were eluted first at retention time 5–11 min and were of cleistanthane, lathyrane, and podocarpane scaffold containing two or three oxygen atoms (peaks 31, 37, 41, 51, 59, 61, 63, and 68, Table 3, Figure 6, Figures S12–S14).

Meanwhile, diterpenoids esters were eluted at retention time 12–22 min and were mostly esters of jatrophane, phorbol, and ingenol. Acetate esters were predominant and were readily identified by the neutral loss of acetic acid (−60 Da) as seen in peaks 67, 70, 94, 112, 113, 114, and 117, while the presence of angelate ester (peak 96) was inferred from the neutral loss of 102 Da. Additionally esterification with benzoic acid was observed in late eluting peaks as indicated by the increase in double bond equivalent (14–16). These were observed in peaks 103 (C_34_H_40_O_9_), 110 (C_38_H_46_O_12_), 113,114 (C_36_H_44_O_10_), 117 (C_36_H_44_O_9_), 123 (C_38_H_48_O_10_), and 126 (C_38_H_48_O_11_) which showed multiple loss of acetate (−60 Da) and fragment ion at *m/z* 123 for benzoic acid, Figures S15 and S16. This substitution pattern of acetate and benzoate esters of a highly oxygenated skeleton has never reported before in *J. integerrima*. However, among Euphorbiaceae plants, molecules with highly oxygenated jatrophane, esterified with benzoic and acetic acid, were isolated from *Pedilanthus tithymaloides* and are designated as peditithin [29], and from *Euphobia sanctae-catharinae* are designated as euphosantiananes, [30,31]. Due to lack of extensive fragmentation of these high molecular weight esters, the exact carbon skeleton of these diterpenes could not be identified.

Among sesquiterpenes annotated in this study, an oxo-hydroxyguai-diene derivative was annotated (C_15_H_22_O_2_, peak 122), which was previously isolated from *J. integerrima* [11]. Two triterpenoids were annotated, namely, oxoamyrin and dioxo-olean-12-ene.

#### 2.4.4. Fatty Acids and Their Conjugates

Fatty acids were detected as free fatty acid, fatty acid glycerides, phospholipids, and glycolipids, which together accounted for 38 metabolites. Unsaturated fatty acids C-18 were most abundant including oxo-phytodienoic (C_18_H_28_O_3_), linolenic (C_18_H_30_O_2_), hydroxylinoleinic (C_18_H_30_O_3_), oxo-octadecadienoic (C_18_H_30_O_3_) and ricinoleic acids. Several fatty acids derivatives were annotated in the extract including monoglyceride (MG), diglycerides (DG), monogalactosyl diglycerides (MGDG), phosphatidic acid esters (PA), and lysophasphatidyl choline (LPC).

Monoglycerides showed two consecutive loss of water (−18 Da), in addition to loss of −92 Da corresponding to loss of glycerol moiety (peaks 82, 108, and 111, Figure S17). Meanwhile, phosphatidic acid esters displayed a characteristic loss of 154 Da and appearance of a fragment ion at 155 amu corresponding to phosphopropionic acid (C_3_H_7_O_5_P) as seen in peaks 80 and 84, Table 3, Figure S18. Annotated phosphatidic acid esters were diglyceride with medium chain length (C-9 and C-10) and were observed in peaks 80 and 84. Fatty acid esters containing choline showed characteristic choline fragment at *m/z* 104.106 as in peaks 86, 87, and 91, Figure S19. Additionally, monogalactosyl diglycerides MGDG were identified in peaks 89, 98, 100, and 109, mainly as monoglycerides of C-16 or C-18 fatty acids.

#### 2.4.5. Phytosterols and Other Compounds

Six phytosterols were annotated in the UPLC/MS chromatogram of *J. integerrima* including *β*-sitosterol, stigmasterol, stigmastane 3,6 dione and stigmast-4-en-6beta-ol-3-one, which all were previously reported in the genus *Jatropha* and the latter was isolated from *J. integerrima.* [11]. Three jasmonates (peaks 36, 39, and 40), two terpene lactones (peaks 17, 18), and tocopherol (peak 133) were also among annotated compounds.

### 2.5. Quantitative Determination of Secondary Metabolites

To gain possible insights into the relative abundance of secondary metabolites in *J. integerrima* extract, spectrophotometric methods were used to estimate the abundance of certain classes of secondary metabolites. Leaves’ extract of *J. integerrima* showed low abundance of phenolic compounds and flavonoids at 70.4 ± 0.4 mg GAE and 10.7 ± 0.1 mg QE per gram of extract, respectively. Meanwhile, results from vanillin/sulfuric assay indicated a high terpenoidal content of 149.7 mg UAE per gram of the extract.

## 3. Discussion

Leaves of *Jatropha integerrima* were used in South-East Asia (particularly in India and Bangladesh) for treatment of some inflammatory conditions such as arthritis and eczema. Other *Jatropha* species have well documented anti-inflammatory activity as proven by folk use, in vitro and in vivo assays [4,32,33]. Our investigation showed that *Jatropha integerrima* leaves extract (JILE) possess an anti-inflammatory effect when used in rat paw edema model. Both oral administration and topical application of the extract were able to reduce signs of inflammation (edema volume) and levels of inflammatory mediators (NO, TNF-α, PKC, and PGE2). Inflammation induced by carrageenan increased paw edema thickness and was associated with elevated levels of NO, TNF-α, PKC, and PGE2 after 4 h, as previously shown in other studies [34]. The involvement of NO and PGs in the modulation of inflammation is well established [35]. Lipid peroxidation initiated by the product of the reaction of NO with superoxide (peroxynitrite) liberates arachidonic acid from the cell membrane activating PGE2, which is one of the strongest inflammatory mediators [36]. Moreover, TNF-α induces NO synthesis by activating inducible nitric oxide synthase iNOS and augments the responses of neutrophils to inflammatory stimuli [37,38]. Activation of PKC mediates the activation of NF-κB, and secretion of TNF-α, IL-6, and IL-10 through TLR2/1 [39]; this may explain the elevation of PKC that provoked the increase in TNF-α serum level in our work.

Treatment with JILE decreased levels of these inflammatory mediators in a dose dependent manner and restored their basal levels at higher treatment dose of 400 mg/kg (per oral route) and using 10% strength cream (after topical application). Additionally, normal tissue structure was observed in animals receiving JILE treatment especially with topical application of 5% or 10% cream or using an oral dose of 400 mg/kg confirming the ability of JILE extract to inhibit inflammatory response.

The metabolomics profile of *J. integerrima* identified many compounds with known anti-inflammatory activity. For example, 19 flavonoidal compounds were detected in the extract which were mainly apigenin derivatives. Apigenin and its C-glycosides (vitexin and isovitexin) are known anti-inflammatory compounds that protect cells against oxidative stress and inflammation [40,41,42]. The major flavonodial compound in the extract (vitexin) was shown to reduce the levels of inflammatory cytokines such as IL4, IL5, and IL13 by 64%, 95%, and 65% in an induced asthma model at concentration of 1 mg/kg [43].

In addition to flavonoids, coumarins such as scopoletin and fraxidin have anti-inflammatory action mediated by their antioxidant potential [44,45,46]. Scopoletin can reduce inflammation and level of PGE2 in LPS stimulated cell lines [47].

Finally, nonpolar compounds such as triterpenes, sterols, and diterpenes are usually main contributors to anti-inflammatory activity of plant extracts. As reported previously, the nonpolar *n*-hexane fraction of *Jatropha curcas* extract had a better anti-inflammatory activity when tested in vitro compared to the polar fraction [33]. Additionally, *J.integerrima* extract was enriched with oxo and hydroxyl fatty acids (13 compounds, Table 3), which received attention recently due to their potential as anti-inflammatory agents and their indigenous role in regulating body inflammatory response [48].

## 4. Conclusions

These results suggest that the ethanol extract of the leaves of *Jatropha integerrima* possess a significant anti-inflammatory effect, both through oral administration and topical application. This effect can be attributed to its high content of flavonoids, terpenoids, and oxygenated fatty acids, which are represented in the metabolomics profile of the extract by 65 compounds. Our findings encourage further investigation of the rich metabolome of *J. integerrima* to identify novel anti-inflammatory compounds.

## 5. Material and Methods

### 5.1. Plant Material

Fresh leaves of *Jatropha integerrima* Jacq. were collected from plants cultivated at the Medicinal, Aromatic and Poisonous Plants Experimental Station of the Faculty of Pharmacy, Cairo University (Giza, Egypt). Plant identity was confirmed by Therese Labib, consultant of plant taxonomy at the Ministry of Agriculture and Orman Botanical Garden (Giza, Egypt). Vouchered herbarium specimen (code numbers 582018III) was deposited at the Pharmacognosy Department, Faculty of Pharmacy, Cairo University.

### 5.2. Preparation of Plant Extract

Fresh leaves were air dried in shade and reduced to powder. The powdered leaves (1 kg) were extracted with absolute ethanol by cold maceration till exhaustion (4 × 5 L). The solvent was then removed by vacuum distillation at a temperature not exceeding 40 °C, and the dried ethanol extract was kept in an air-tight container at 4 °C till use.

### 5.3. Determination of Total Phenolic Content

Total phenolic content of *J. integerrima* leaves extract was carried out using the Folin–Ciocalteu method following the optimized assay procedures described by Blainski, Lopes, and de Mello [49]. The total phenolic content was expressed as mg gallic acid equivalent (GAE)/g of extract using a standard calibration curve of gallic acid (20–200 μg/mL).

### 5.4. Determination of Flavonoid Content

Total flavonoid content was determined by measuring the yellow color developed upon reacting flavonoids with AlCl_3_ reagent according to optimized assay conditions described by Silva et al. [50]. The total flavonoid content was expressed in mg quercetin equivalent (QE)/g of extract based on pre-established calibration curve of quercetin (0.1–0.7 mg/mL).

### 5.5. Determination of Steroidal/Terpenoidal Content

Spectrophotometric estimation of total steroidal and/or terpenoid content was carried out based on the chromogenic reaction produced upon treatment of the extract with vanillin/ sulfuric acid reagent according to the protocol developed by V. Le et al. [51]. The color developed was measured at λ = 544 nm, and total steroid and/or terpenoid content was expressed as ursolic acid equivalent (UAE mg/g of extract) as deduced from a pre-established calibration curve using standard ursolic acid (20–160 μg/mL).

### 5.6. Ultra-Performance Liquid Chromatography Analysis

Liquid chromatography instrument (Sciex, TripleTOF 5600+, Framingham, MA, USA) was used for chromatographic separation. A reversed phase C18 analytical column (Waters, Xbridge C18, 2.1 × 50 mm, 3.5 µm particle size) was used at 40 °C. Leaves extract (50 mg) was dissolved in 1 mL of water: methanol: acetonitrile (50:25:25) and centrifuged for 5 min at 10,000 rpm followed by filtration through a membrane disc filter. Twenty microliters were diluted to 1 mL and 10 µL of this solution was injected into the system. Gradient elution was carried out at a flow rate of 0.3 mL/min using eluent A (0.1% formic acid in deionized water) and eluent B (100% acetonitrile). Elution was performed according to the following gradient: 10% B, 0–1 min; 10%–90% B, 1–25 min; 90%–10% B, 25–28 min.

### 5.7. High Resolution Quadrpole-Time of Flight Mass Analysis

UPLC system was coupled to electrospray ion source with quaderpole-time of flight mass analyzer (ESI-QTOF, Framingham, MA, USA). MS analysis was performed in positive ion mode; cone voltage, 30 eV; capillary voltage, 3 kV; desolvation temperature, 450 °C; cone gas flow, 50 L/h and desolvation gas flow, 900 L/h. Time of flight mass scan (TOF-MS, Framingham, MA, USA) was controlled using Analyst TF 1.7.1 and was recorded over *m/z* range 50–1000. TOF MS/MS scan was done in information dependent acquisition over the same mass range. UPLC-MS/MS data were processed with PeakView 2.2 (Framingham, MA, USA) for data extraction.

### 5.8. Anti-Inflammatory Assay

#### 5.8.1. Animals

Adult male Wister albino rats (7–8 weeks old, weighing 130–180 g) were housed in standard cages (10 rats/cage) under pathogen-free conditions and maintained at controlled room temperature (21–24 °C) with a 40–60% relative humidity and under normal dark-light cycles. All animals had free access to rat chew diet and water *ad libitum*. All procedures were approved by the Animal Care Committee of the National Research Centre.

#### 5.8.2. Acute Toxicity Study

Rats were divided into two groups of 12 rats each (6 males and 6 females). The extract of *J. integerrima* was suspended in distilled water and given orally to rats of the first group in a single dose of 5 g/kg. The control group received the same volumes of distilled water. The percentage mortality for the extract was recorded for 24 h. Animals were observed for 14 days, for any changes in skin, fur, respiratory, circulatory, central nervous system, somatomotor activity, and behavior pattern. Particular observation for tremors, convulsions, salivation, diarrhea, lethargy, sleep, and coma were also recorded.

#### 5.8.3. Carrageenan Induced Paw Edema

Anti-inflammatory activity was evaluated using carrageenan induced paw edema. Paw swelling was elicited by subplantar injection of 100 μL of 1% sterile lambda carrageenan suspension in saline into the right hind paw [35]. Contralateral paw received an equal volume of saline solution. The edema component of inflammation was quantified by measuring hind footpad immediately before carrageenan injection and 1–4 h after carrageenan injection with a micrometer caliber [52]. Edema was expressed as a percentage of change from control (predrug) values. Rats, to be injected with carrageenan, were divided into nine groups each of 10 animals. Group 1 (control group): where rats received oral saline (0.2 mL/rat); group 2: rats were given indomethacin (25 mg/kg); groups 3 and 4: rats received the extract of *J. integerrima* (200 mg/kg and 400 mg/kg), respectively. Indomethacin and the extracts were given orally 60 min before the injection of the carrageenan suspension.

#### 5.8.4. Skin Irritation Test

Twelve rats were divided into two groups (six rats each), namely, control and test groups. Hairs of rat hind paw were shaved, and rats of different groups were kept in separate cages for seven days. An amount (0.3 g) of 10% cream was placed on the shaved skin (4 cm^2^) for rats of the test group while the other group received only base cream. The area was covered with a cotton bandage and any sensitivities were assessed and graded.

#### 5.8.5. Topical Anti-Inflammatory Activity

The base cream was prepared according to recipe by Franyoto et al.( 2018) [53] containing stearic acid (142 g), glycerine (100 g), sodium tetraborate (2.5 g), triethanolamine (10 g), methyl paraben (0.1 g) and 750 mL of distilled water. The base cream was then mixed with the ethanol extract of the leaves of *Jatropha integerrima* at three different concentrations (2.5%, 5%, and 10%) [53].

Before edema induction as described in Section 5.8.3, rats were divided into six groups each of 10 rats. Group 1 was left untreated, while animals in group 2 were given a single topical dose (0.3 g) of market product of hydrocortisone (1%). Group 3 rats received a single topical dose (0.3 g) of base cream, while animals in groups 4, 5, and 6 received 0.3 g of the cream extract of *J. integerrima* at concentrations of 2.5%, 5%, and 10%, respectively. An amount of 0.3 g of the tested cream was used 30 min before injection of the carrageenan suspension, and it was gently rubbed 50 times with the index finger. A separate group of healthy animals were kept as a control group. Edema was expressed as a percentage change from control (predrug) values [54].

#### 5.8.6. Blood Samples and Biochemical Analysis

Four hours after carrageenan injection and immediately after measuring edema volume, animals were anesthetized with urethane (1.5 g/kg; i.p.) and blood samples were taken from the abdominal aorta and used for determination of PGE2 using Abnova ELISA Kit, TNF-α using Cusabio ELISA Kit, and PKC by enzyme linked immunoassay (ELISA) technique using standard kits (Glory Science Co., Ltd, Louisiana, LA, USA).

#### 5.8.7. Histological Examination

Paws were fixed in 10% formalin solution and dehydrated in ascending grades of alcohol and embedded in paraffin. Sections at four-micron thickness were taken and stained with hematoxylin and eosin (H&E).

### 5.9. Statistical Analysis

All the values are presented as mean ± standard error of the means (SE). Comparisons between different groups were carried out using one-way analysis of variance (ANOVA), followed by Tukey’s HSD test for multiple comparisons. Graph pad Prism software, version 5 (GraphPad Software Inc., San Diego, CA, USA), was used to carry out statistical tests. The difference was considered significant when *p* < 0.05.

## Figures and Tables

**Table 1:** Effect of oral administration of *Jatropha integerrima* leaves extract (JILE) on different inflammation biomarkers.

	Control	Carrageenan	Indomethacin 25 mg/kg	JILE 200 mg/kg	JILE 400 mg/kg
NO (µmol/L)	16.00 ± 1.29	23.00 ± 0.676 ^a^	19.03 ± 0.3 ^ab^	19.9 ± 0.08 ^ab^	18.55 ± 0.39 ^b^
PGE2 (pg/mL)	251.28 ± 4.16	498.8 ± 37.04 ^a^	354.88 ± 5.36^ab^	514.88 ± 5.36 ^a^	352.4 ± 23.52 ^ab^
TNF-*α* (pg/mL)	1581.92 ± 10.88	2194.56 ± 99.52 ^a^	1804.8 ± 7.84 ^ab^	2104.96 ± 4.16 ^a^	1823.68 ±0.16 ^ab^
PKC (pg/mL)	870.4 ± 99.705	1505.35 ± 5.78 ^a^	842.35 ± 153.85 ^b^	805.8 ± 62.985 ^b^	785.4 ± 23.29 ^b^

Data were expressed as mean ± SD. Statistical analysis was carried out by one-way ANOVA, followed by Tukey’s HSD test for multiple comparisons. ^a^: significantly different from normal control (Saline), ^b^: significantly different from carrageenan control at *p* < 0.05.

**Table 2:** Effect of topical administration of ethanol extract of *Jatropha integerrima* cream on different inflammation biomarkers.

	Control	Carrageenan	Hydrocortisone 1% Cream	Base	JILE 2.5% Cream	JILE 5% Cream	JILE 10% Cream
NO (µmol/L)	17.9 ± 0.7	25 ± 0.7 ^a^	21.29 ± 0.6 ^ab^	24.31 ± 0.1 ^a^	22.27 ± 0.59 ^ab^	20.74 ± 0.58 ^ab^	20.49 ± 0.08 ^ab^
PGE2 (pg/mL)	215.4 ± 1.5	286.2 ± 0.4 ^a^	223.4 ± 0.2 ^b^	284.4 ± 1.8 ^a^	256.4 ± 1.4 ^ab^	243.4 ± 1.1 ^ab^	216.4 ± 1.1 ^b^
TNF-α (pg/mL)	1561.9 ± 13.6	1903.9 ± 62.9 ^a^	1615.9 ± 15.68 ^b^	1857.9 ± 78.1 ^a^	1804.5 ± 23.7 ^a^	1779.9 ± 4.4 ^ab^	1609.9 ± 22.3 ^b^
PKC (pg/mL)	854.9 ± 22.8	1259.9 ± 4.4 ^a^	854.98 ± 15.1 ^b^	1251.9 ± 26.4 ^a^	965.98 ± 9.8 ^ab^	924.9 ± 11.1 ^ab^	839.9 ± 2.2 ^b^

Data were expressed as mean ± SD. Statistical analysis was carried out by one-way ANOVA followed by Tukey’s HSD test for multiple comparisons. ^a^: Significantly different from normal control (Saline) at *p* < 0.05. ^b^: Significantly different from carrageenan control at *p* < 0.05.

**Table 3:** Annotated metabolites in leaves extract of *Jatropha integerrima* using UPLC/MS-Q-TOF analysis.

Peak No.	tR (min)	Experimental *m*/*z* [M + H]^+^	Molecular Formula	Error (ppm)	Fragments (*m*/*z*)	Tentative Identification
**Flavonoids**						
10	0.981	595.1656	C_27_H_30_O_15_	3.4	577, 433, 379, 367, 313, 283	Isovitexin-*O*-hexoside
11	2.68	449.1076	C_21_H_20_O_11_	2.3	431,383, 353, 339, 329, 299	Isoorientin
13	2.915	433.1116	C_21_H_20_O_10_	3.2	415, 379, 367, 337, 313, 283	Vitexin
14	3.13	433.1116	C_21_H_20_O_10_	3.2	415, 379, 337, 323, 313, 283	Isovitexin
16	3.4	449.1076	C_21_H_20_O_11_	2.9	431, 395, 287	Kaempferol *O* hexoside
19	3.67	560.1788	C_27_H_29_NO_12_	4.5	445, 427,409, 325, 295	Methylisovitexin proline
20	3.912	595.1672	C_27_H_30_O_15_	2.2	287, 271	Kaempferol *O*-rutinoside
22	4.05	771.2151	C_37_H_38_O_18_	2.6	675, 651,433,415, 313, 283, 177	Vitexin ferulate-*O*-hexoside
24	4.123	771.2111	C_37_H_38_O_18_	−2.6	675, 651,433,415, 313, 283, 177	Isovitexin ferulate hexoside
25	4.253	579.1708	C_27_H_30_O_14_	−0.4	433,417,399,351,321 297, 271	Apigenin *O*-rhamnoside-*O*-hexoside
26	4.396	433.1155	C_21_H_20_O_11_	4.6	271	Apigenin-*O*-hexoside
27	4.44	579.1717	C_27_H_30_O_14_	−1.5	271.0597	Apigenin-*O*-rutinoside
29	5.213	553.1329	C_28_H_24_O_12_	−1	433, 415,397,337,313, 295, 283,	Vitexin *p* hydroxy benzoate
30	5.253	877.2175	C_30_H_40_O_20_	1.2	859, 757, 739, 455, 379, 325	Jatrophenol I/II/III
32	5.942	877.219	C_30_H_40_O_20_	0.5	859, 445, 427, 409,379,349,325	Jatrophenol I/II/III
33	6.06	639.1772	C_32_H_30_O_14_	2.1	415, 271,207	Apigenin sinapoyl hexose
34	6.13	609.159576	C_31_H_28_O_13_	2.4	489, 433, 397, 313, 283, 177	Apigenin ferulate-*C*-hexoside
35	6.215	736.2208	C_33_H_36_NO_15_	3.8	621,585,427,391,325, 295,177	Methylisovitexin proline ferulate ester
58	11.1	225.091	C_15_H_12_O_2_	0.0	197, 165, 105	Flavanone
**Phenolic Acid Conjugates**						
21	3.98	417.1347	C_18_H_24_O_11_	−4.4	237,109	Cinnamic glycerol hexoside
23	4.06	503.1766	C_22_H_30_O_13_	1.4	485, 467, 383	Methyl-O-feruloylquinate diacetate
52	9.9	261.1852	C_17_H_24_O_2_	1.1	243, 201, 147, 119	Octyl cinnamic acid
56	10.57	221.1537	C_14_H_20_O_2_	5.0	203, 161, 151, 133, 123, 105	4-Heptylbenzoic acid
74	13	291.1958	C_18_H_26_O_3_	1.1	193, 123	Octyl-4-methoxycinnamate
76	13.1	235.17	C_15_H_22_O_2_	3.2	179,123,57	Octyl benzoate
106	19.5	401.3422	C_27_H_44_O_2_	2.0	191, 177, 137	Eicosenoyl benzoate
118	21.4	391.2837	C_24_H_38_O_4_	1.2	167,149	Tetradecyl ferulate
128	22.17	419.3153	C_26_H_42_O_4_	0.7	275, 293, 275,177, 127	Hexadecyl ferulate
129	23.4	463.3793	C_29_H_50_O_4_	2.4	445, 417, 177,139	Nonadecyl ferulate
**Coumarins**						
12	2.9	193.0492	C_10_H_18_O_4_		137, 77,53	Scopoletin
15	3.31	223.0595	C_11_H_10_O_5_	2.7	149, 207, 121	Fraxidin
28	5.081	501.159	C_22_H_28_O_13_	2.5	339, 321, 177, 209	Methylhydroxycoumarin dihexoside
42	9.2	223.0738	C_15_H_10_O_2_	5.7	177, 149, 121	4-Phenyl coumarin
**Nitrogenous Compounds**						
1	0.465	266.1604	C_11_H_23_NO_6_	2.2	248, 230, 116,104, 87	Choline hexoside
4	0.515	104.106585	C_5_H_13_NO	0.9	56.04, 58.06, 59.07, 60.07, 71	Choline
5	0.515	116.070497	C_5_H_9_NO_2_	0.9	70	L-Proline
6	0.541	144.100864	C_7_H_13_NO_2_	0	58, 84	Stachydrine
7	0.6	138.0549	C_7_H_7_NO_2_	0.4	136, 94,92,79,66	Amino benzoic acid
8	0.65	213.1241	C_10_H_16_N_2_O_3_	2.3	195, 177,135,121	Prolylproline
9	0.975	146.060114	C_9_H_7_NO	1.0	51, 65, 77, 91, 117, 118, 128	Indole carboxyaldehyde
73	12.96	270.3153	C_18_H_39_N	0.8	158	Octadecylamine
**Cyclic Peptides**						
43	9.51	722.4826	C_36_H_63_N_7_O_8_	1.3	623, 510, 379, 280, 183	Cyclo (Val-leu-Leu-Val-Ser-Leu-Pro)
45	9.59	809.5482	C_40_H_72_N_8_O_9_	−1.8	722, 623, 605, 510, 280, 211	Cyclo (ABA-Val-leu-Leu-Val-Ser-Leu-Val)
46	9.6	767.5383	C_38_H_70_N_8_O_8_	−1.5	722, 704, 623, 510, 379	Cyclo (MeLys-leu-Leu-Val-Ser-Leu-Val)
47	9.7	652.4030	C_31_H_53_N_7_O_8_	0.24	634,521, 381, 268, 181	Integerrimacyclopeptide B
49	9.84	782.4596	C_39_H_59_N_9_O_8_	4.8	669, 651, 355, 284	Cyclogossine A
53	10.05	781.4595	C_40_H_60_N_8_O_8_	1.5	763, 668, 650, 555, 468	Integrimide A
54	10.53	767.5031	C_37_H_66_N_8_O_9_	0.7	749, 654, 636, 523, 394	Integerrimacyclopeptide A
55	10.55	668.430	C_32_H_57_N_7_O_8_	−3.2	650, 555, 537, 424,367,284,171	Cyclo (Leu-Leu-Gly-Thr-Leu-Ala-Val)
**Diterpenes**						
31	5.814	317.2112	C_20_H_28_O_3_	0.6	299, 271, 231, 175, 173	Cleistanthol/ Jatrointelone C
37	6.88	291.1951	C_18_H_26_O_3_	1.3	255, 167	Triihydroxy-13- methylpodocarpane-triene
41	9.11	317.2110	C_20_H_28_O_3_	−0.06	299, 271,231,175	Jatrointelone C/ Cleistanthol
51	9.9	275.2008	C_18_H_26_O_2_	0.88	257,239, 173,159,119	Dihydroxy-13- methylpodocarpane-triene
59	11.36	301.21539	C_20_H_28_O_2_	2.7	283 239 227 218 185	Spruceanol
61	11.95	275.2016	C_18_H_26_O_2_	3.8	257, 173, 131, 91	Dihydroxy methylpodocarpane-8,10,13-triene isomer
63	11.98	315.1936	C_20_H_26_O_3_	5.9	231, 199, 133,123,81	Multifidone
67	12.4	411.2355	C_22_H_34_O_7_	5.4	351, 333, 369	Excolabdone C/ Isoforskolin
68	12.6	289.179	C_18_H_24_O_3_	−2.8	221, 205	Methyl podocarpate
70	12.7	671.3041	C_36_H_46_O_12_	−3.1	621, 593, 331	Premyrsinol propanoate-benzoate-triacetate
90	15.8	321.2428	C_20_H_32_O_3_	1.2	275, 257	Jatrodagricaine A
94	17.57	609.2703	C_34_H_40_O_10_	0.5	591, 531, 515, 273, 123	Diterpene benzoate triacetate
96	18	415.2391	C_25_H_34_O_5_	1.8	369, 313, 295	Deoxyingenol angelate
101	18.18	651.2815	C_36_H_42_O_11_	1.5	633, 601, 573, 483, 283	Diterpene benzoate tetracetate
103	18.65	593.2741	C_34_H_40_O_9_	−0.7	547, 533, 461, 447	Diterpene benzoate triacetate
104	18.85	575.3942	C_34_H_54_O_7_	0.1	309, 177	Phorbol-12-Myristate
110	20.15	695.307	C_38_H_46_O_12_	1.1	649, 563, 517	Diterpene benzoate pentacetate
112	20.37	653.2981	C_36_H_44_O_11_	2.4	609, 575,565, 549,503, 521	Euphorbiaproliferin F or isomer
113	20.4	637.3002	C_36_H_44_O_10_	−1.6	619, 587, 559	Peditithin H or isomer
114	20.52	637.3018	C_36_H_44_O_10_	0.8	619, 587,559, 473	Peditithin H or isomer
117	21.1	621.3045	C_36_H_44_O_9_	−1.8	561, 533,461,433, 193	Diterpene benzoate triacetate
119	21.46	431.2432	C_25_H_34_O_6_	0.9	231,165	Ingenol mebutate
123	21.9	665.3349	C_38_H_48_O_10_	4.3	619, 587, 559	Diterpene dibenzoate diacetate
126	22.03	681.3265	C_38_H_48_O_11_	−0.6	635, 593, 549, 503	Diterpene dibenzoate diacetate
**Sesquiterpenoids**						
57	10.9	219.1751	C_15_H_22_O	3.0	203	Unidentified sesquiterpenoid
60	11.6	253.179	C_15_H_24_O_3_	3.2	197, 179, 141, 151	Ilicic Acid
75	13.06	235.1700	C_15_H_22_O_2_	3.2	179, 123,57	4-Patchoulen-15-oic acid
122	21.65	235.1697	C_15_H_22_O_2_	1.9	179, 81	Oxo-hydroxyguai-diene
**Triterpenoids**						
107	20.03	441.3736	C_30_H_48_O_2_	2	423 287 235 189 149	Oxoamyrin
116	20.9	439.3573	C_30_H_46_O_2_	0.6	301, 233, 173, 149, 121	Dioxo-olean-12-ene
**Fatty Acids and Their Conjugates**						
38	7.46	293.2125	C_18_H_28_O_3_	4.7	275, 257, 213, 195, 155	Oxo-phytodienoic acid
44	9.53	767.5324	C_43_H_74_O_11_	2.6	623, 511	GlcADG (34:3)
62	11.95	293.2113	C_18_H_28_O_3_	0.6	275, 257, 147, 133	Oxo-phytodienoic acid
64	12.26	319.2256	C_20_H_30_O_3_	−5.3	301	Oxo-eicosatetraenoic acid
65	12.4	313.2362	C_18_H_32_O_4_	3.6	295,277,151,95,81	Hydroperoxy-octadecadienoic acid
66	12.4	295.2278	C_18_H_30_O_3_	3.5	277,179,149,151, 137, 119	Oxo-octadecadienoic acid
69	12.63	277.2166	C_18_H_28_O_2_	1.4	259, 135,149, 121, 107	Octadecatetraenoic acid
71	12.85	295.2276	C_18_H_30_O_3_	2.8	277, 259, 231, 165	Hydroxy-Octadecatrienoic
72	12.86	351.2539	C_21_H_34_O_4_	2.9	277, 259, 179, 149,133	MG 18:4
77	13.48	520.3395	C_26_H_50_NO_7_P	−0.9	502,184,104	LPC 18:2
78	13.5	295.2278	C_18_H_30_O_3_	6.2	277,179,149,151, 137,119	Hydroxy linoleinc acid
79	13.53	279.232	C_18_H_30_O_2_	0.5	149, 95, 81	Linolenic acid
80	13.7	465.2623	C_22_H_41_O_8_P	1.22	447,311,237, 155	PA: (10:1/ 9:0)
81	13.76	313.2734	C_19_H_36_O_3_	1.0	295, 277, 165, 123, 95	Oxononadecanoic acid
82	13.8	331.2899	C_19_H_38_O_4_	1.9	313, 257, 239, 123	2-Monopalmitin MG 16:0
83	13.94	295.2278	C_18_H_30_O_3_	3.5	277,179,149,151, 137, 119	Hydroxy-octadecatrienoic acid
84	14.08	467.2784	C_22_H_44_O_8_ P	2.17	393,313,239,155	PA (10:0/ 9:0)
85	14.1	295.2265	C_18_H_30_O_3_	0.9	277, 179, 151	Hydroxylinolenic acid
86	14.18	496.3403	C_24_H_50_NO_7_P	−0.34	478,184,125, 104, 86	LPC 16:0
87	14.3	522.3580	C_26_H_52_NO_7_P	3.89	504,184,150, 104	LPC 18:1
88	15.3	291.1945	C_18_H_26_O_3_	3.3	273 249 203 147	Oxo-octadecatetraenoic acid
89	15.37	723.5084	C_41_H_70_O_10_	5.2	177, 133,89	MGDG 16:2, 16:2
91	16.095	524.3709	C_26_H_54_NO_7_P	−1.36	506,184,104	LPC 18:0
92	16.19	305.2483	C_20_H_32_O_2_	2.6	259, 149, 135	Arachidonic acid
93	17.1	307.2631	C_20_H_34_O_2_	0.4	329, 307	Eicosatrienoic acid
95	17.64	323.2590	C_20_H_34_O_3_	2.0	305, 277, 179,151	Hydroxylinoleinic acic ethyl ester
97	18.06	331.2846	C_19_H_38_O_4_	0.9	313, 257, 239	1-Monopalmitin MG 16:0
98	18.07	699.5008	C_39_H_70_O_10_	4.6	625, 607, 429	MGDG 16:2, 14:0
99	18.07	353.2665	C_21_H_36_O_4_	4.0		2-Monolinolenin MG 18:3
100	18.08	745.483	C_43_H_68_O_10_	−5.5	699, 625, 415, 295	MGDG 16:3, 18:4
102	18.24	782.5670	C_44_H_80_NO_8_P	−3.8	765,307	Phosphatidylcholine (18:2/18:2)
108	20.1	359.3145	C_21_H_42_O_4_	3.0	341, 285, 267, 123	Monosteirin
109	20.13	755.5713	C_43_H_78_O_10_	3.9	681,663,443,323	MGDG 18:2/16:0
111	20.15	381.2996	C_23_H_40_O_4_	0.9	none	MG 20:3
115	20.89	549.4527	C_34_H_60_O_5_	2.5	531, 513, 287, 189, 121	DG 14:1/17:2
121	21.6	313.2723	C_19_H_36_O_3_	4.5	295, 277, 149, 133	Ricinoleate methyl esters
125	22	613.4828	C_39_H_64_O_5_	0.2	595, 335, 261	Dilinolenin DG 18:3/18:3
132	25.1	591.4920	C_37_H_66_O_5_	−6.3	573, 335,313,261	DG 18:3/16:0
**Phytosterols**						
105	19.36	445.3630	C_29_H_48_O_3_	−4.6	427, 341, 185	Oxo-hydroxy sitosterol
120	21.55	413.3792	C_29_H_48_O	2.07	395, 159	Stigmasterol
124	21.9	429.3723	C_29_H_48_O_2_	0.9	411	Stigmast-4-en-6beta-ol-3-one
127	22.15	461.3616	C_29_H_48_O_4_	2.0	443, 401, 383, 187	Trihydroxystigmastan-6-one ene
130	23.5	415.3964.8	C_29_H_50_O	4.8	397, 341	Sitosterol
131	23.7	429.3729	C_29_H_48_O_2_	0.2	219, 205, 165	Stigmastane 3,6 dione
**Jasmonates**						
36	6.263	265.1433	C_15_H_20_O_4_	0.5	247, 219,207,205,167,99 191	Abscisic acid
39	7.55	181.122163	C_11_H_16_O_2_	9.4	163,135, 121, 107, 99, 93	Jasmorolone
40	8.6	255.1498	C_13_H_20_O_3_	5.7	195, 179, 143,137	Methyljasmonate
**Miscellaneous Compounds**						
2	0.47	365.1069	C_14_H_20_O_11_	2.6	203, 185	Ethyl aconitate hexoside
3	0.48	527.1584	C_20_H_30_O_16_	4.8	365, 347, 203, 185	Ethyl aconitate dihexoside
17	3.56	197.1168	C_11_H_16_O_3_	−2.1	179,161,135, 107	Loliolide/Epiloliolide
18	3.63	197.1170	C_11_H_16_O_3_	−1.2	179, 161, 133, 105, 91	Loliolide/Epiloliolide
48	9.8	409.166	C_24_H_24_O_6_	3.5	289, 121, 119	Benzyl shikonin
50	9.88	387.1793	C_22_H_26_O_6_	−2.3	267, 147, 121, 105	Eudesmin/epieudesmin
133	25.45	417.3652	C_28_H_48_O_2_	0.3	191, 151	Tocopherol

DG: diglyceride; LPC: lysophosphatidylcholine; MG: monoglyceride; MGDG: monoglyceridedi galactoside; PA: phosphatidic acid.

## Data Availability

All data related to this article are presented in this manuscript or available as supplementary material.

## Supplementary Materials

The following are available online at https://www.mdpi.com/article/10.3390/plants11020218/s1, Figure S1: MS/MS spectrum of peak 29: Vitexin-*p*-hydroxybenzoate; Figure S2: Fragmentation pattern highlighting different fragmentation of the two structural isomer vitexin and isovitaxin based on the intensity of 313 and 283 fragment ions; Figure S3: MS/MS spectrum of peaks 30 and 32: Jatrophenol I/II/III; Figure S4: MS/MS spectrum of peak 35: Proline methylisovitexin; Figure S5: MS/MS spectrum of peak 35: Methylisovitexin proline ferulate; Figure S6: MS/MS spectrum of peak 26: Apigenin-*O*-hexoside; Figure S7: MS/MS spectrum of peak 25: Apigenin-*O*-hexoside-*O*-rhamnoside; Figure S8: MS/MS spectrum of peak 43: A cycloheptapeptide; Figure S9: MS/MS spectrum of peak 45: A cyclooctapeptide; Figure S10: MS/MS spectrum of peak 46: A cycloheptapeptide; Figure S11: MS/MS spectrum of peak 46: A cycloheptapeptide; Figure S12: MS/MS spectrum of peak 59: Spruceanol; Figure S13: MS/MS spectrum of peak 61; Figure S14: MS/MS for peak 67: Isoforskolin; Figure S15: MS/MS for peak 114: Premyrsinol/peditithin derivatives; Figure S16: MS/MS of peak 125: Premyrsinol/peditithin derivative; Figure S17: MS/MS of peak 82: Monopalmitin; Figure S18: MS/MS of peak 84: Phosphatidic acid (10:0/9:0); Figure S19: MS/MS of peak 86: Lysophosphatidylcholine (16:0/0:0); Table S1: Analysis of high resolution MS/MS Q-TOF fragments for newly identified cyclic peptides.
