# Supplementary material for: Oral and Topical Anti-Inflammatory Activity of Jatropha integerrima Leaves Extract in Relation to Its Metabolite Profile
Source: Plants (Basel). 2022 Jan 14;11(2):218. doi: 10.3390/plants11020218 (PMC8781579; doi:10.3390/plants11020218)
Supplement: Supplementary file 1 [file plants-11-00218-s001.zip › plants-1512158-supplementary.pdf]

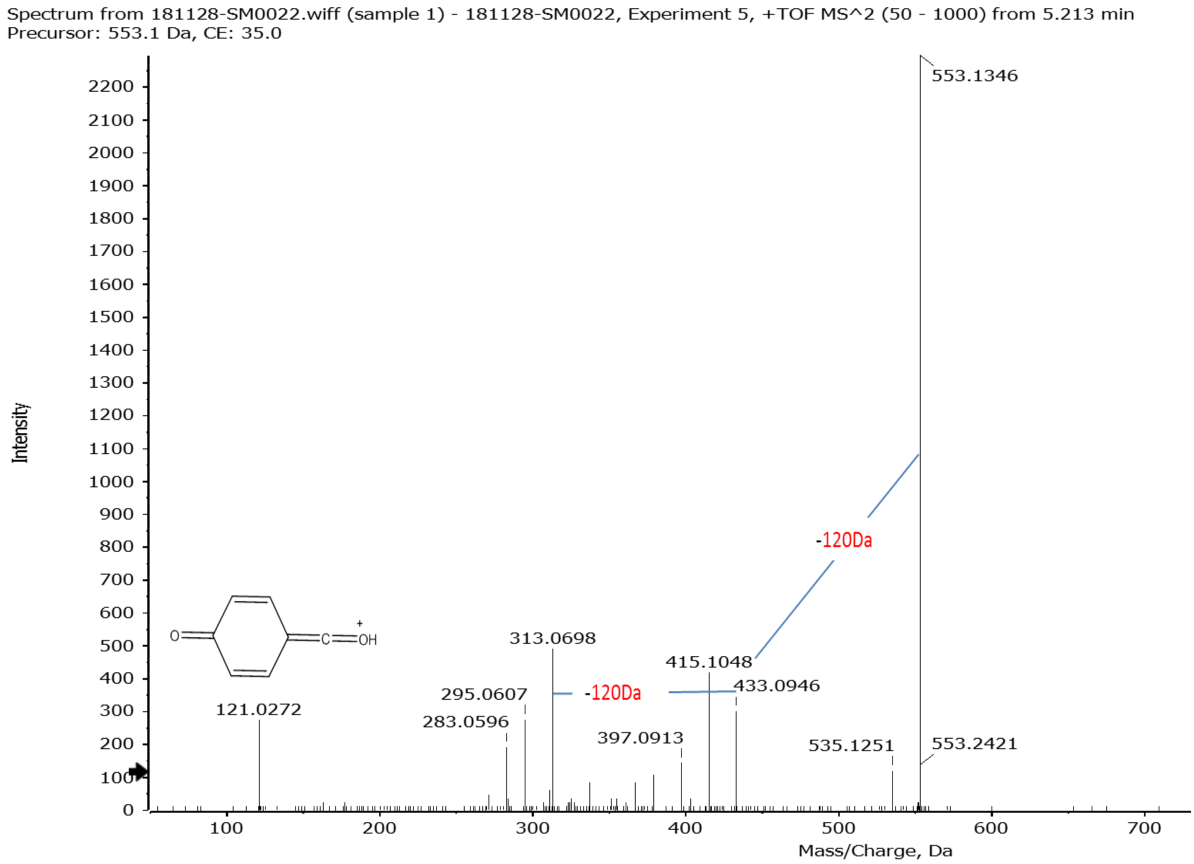

Figure S1. MS/MS spectrum of peak 29: Vitexin-p-hydroxybenzoate.

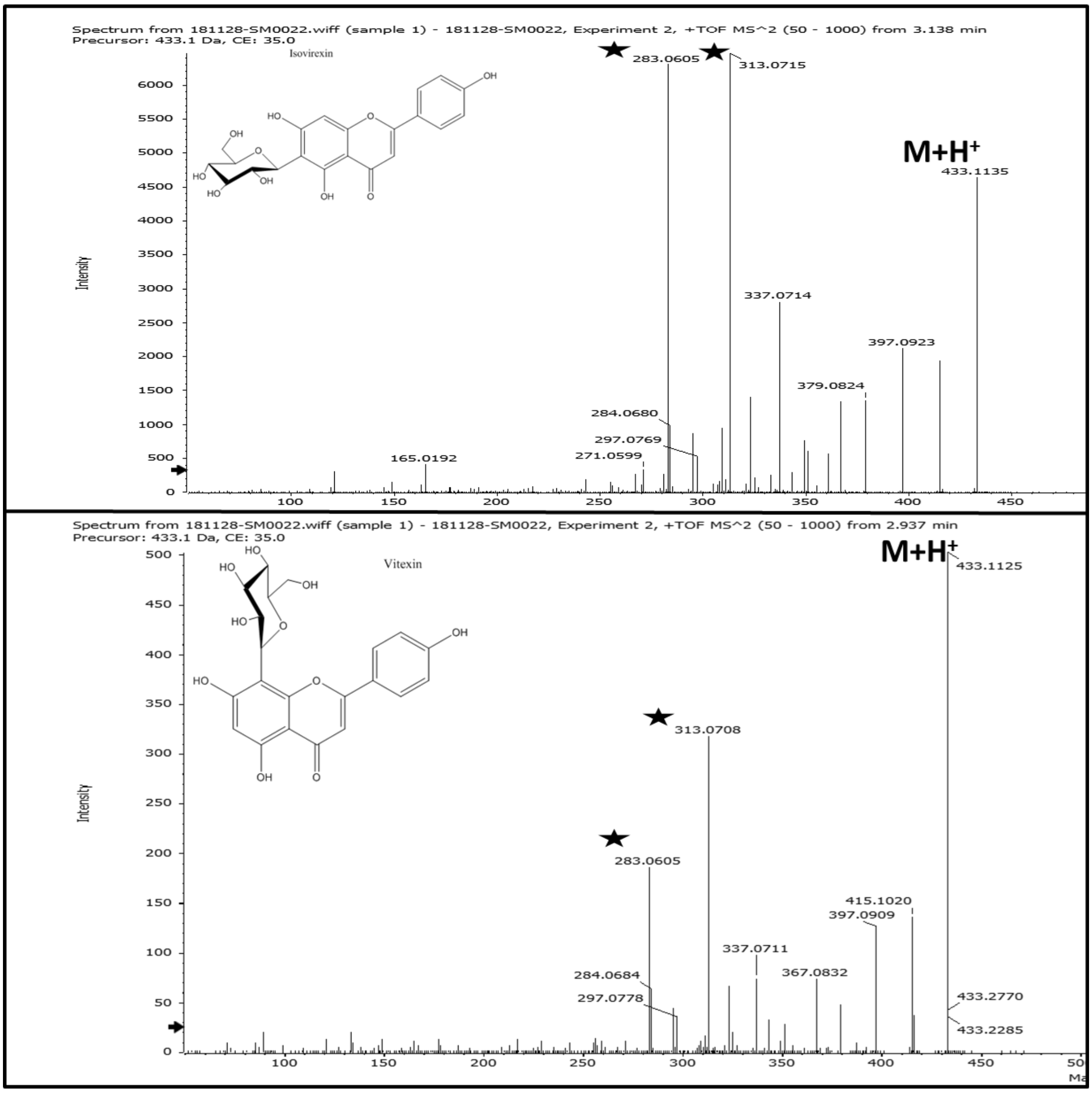

Figure S2. Fragmentation pattern highlighting different fragmentation of the two structural isomer vitexin and isovitaxin based on the intensity of 313 and 283 fragment ions.

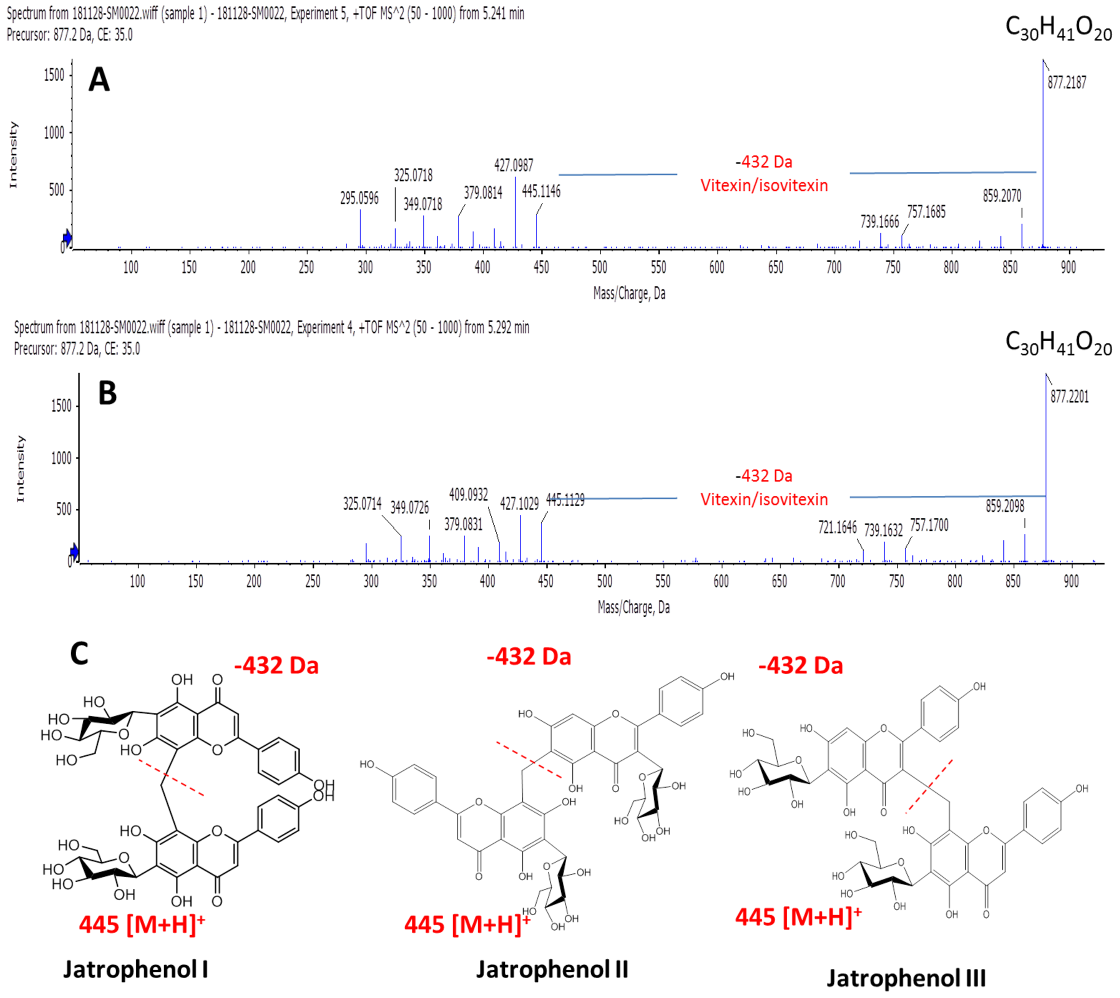

Figure S3. MS/MS spectrum of peaks 30 and 32: Jatrophenol I/II/III.

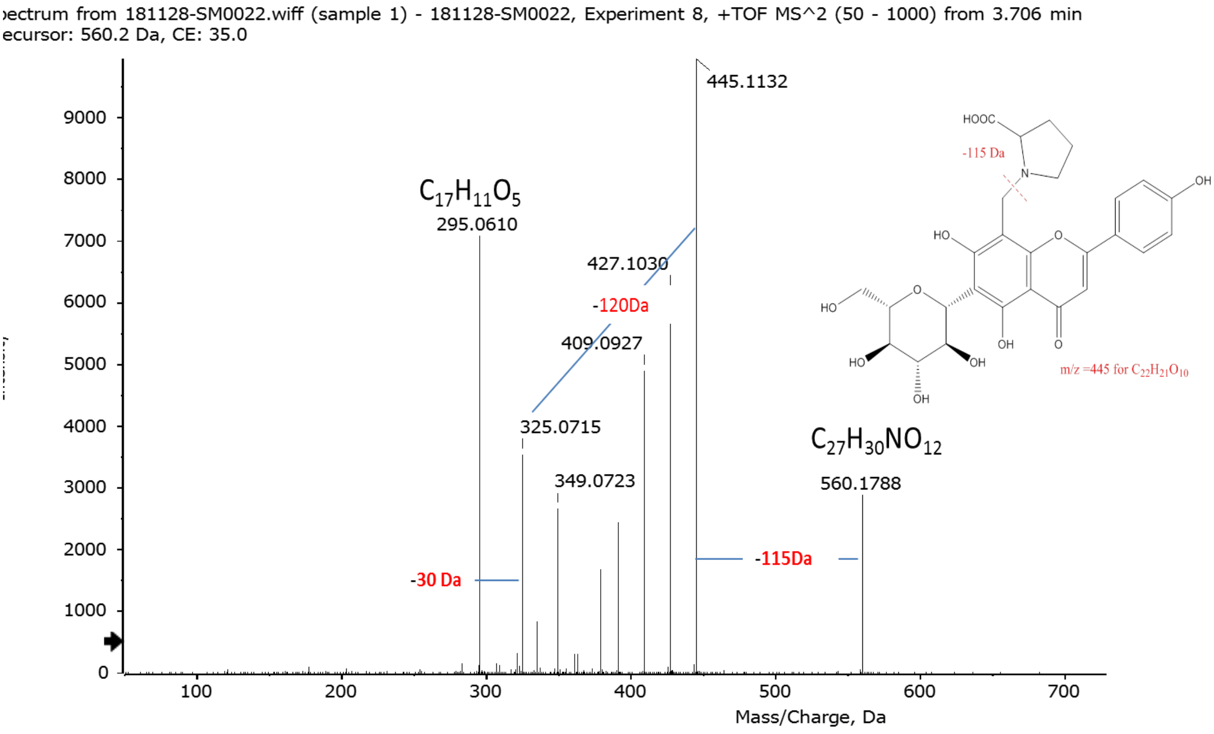

Figure S4. MS/MS spectrum of peak 35: Proline methylisovitexin.

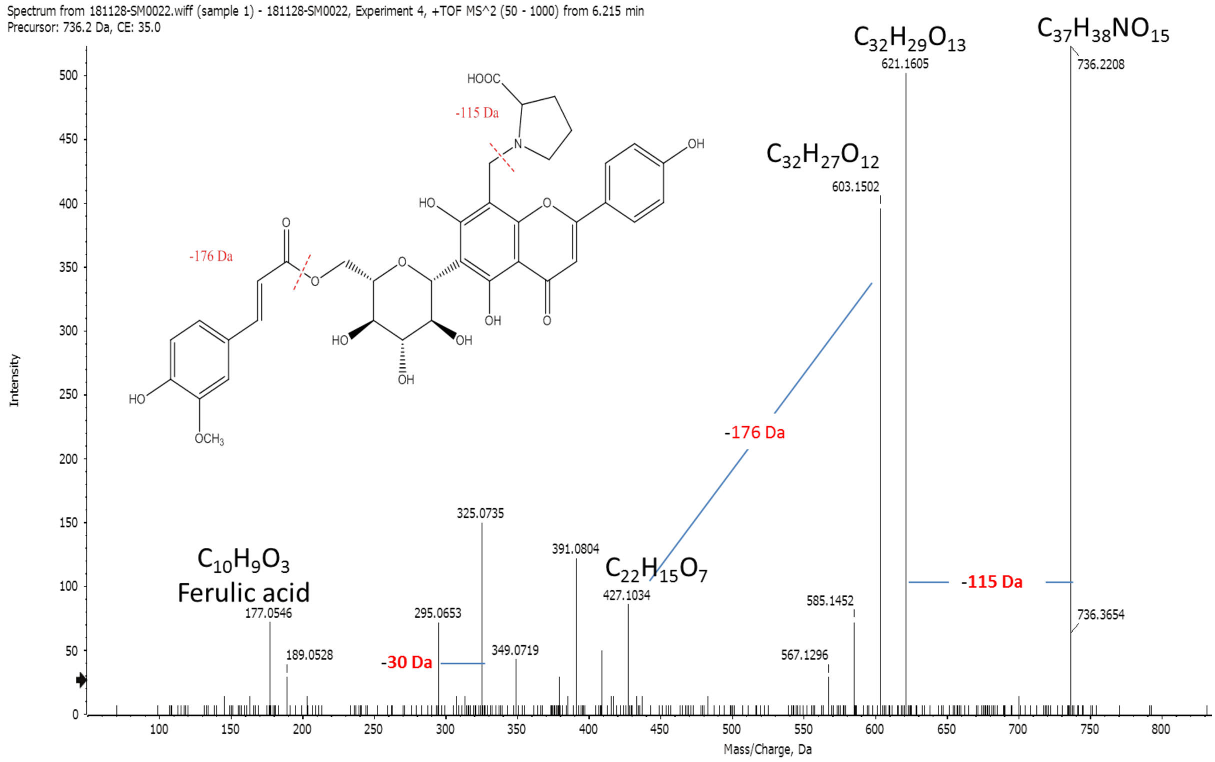

Figure S5. MS/MS spectrum of peak 35: Methylisovitexin proline ferulate.

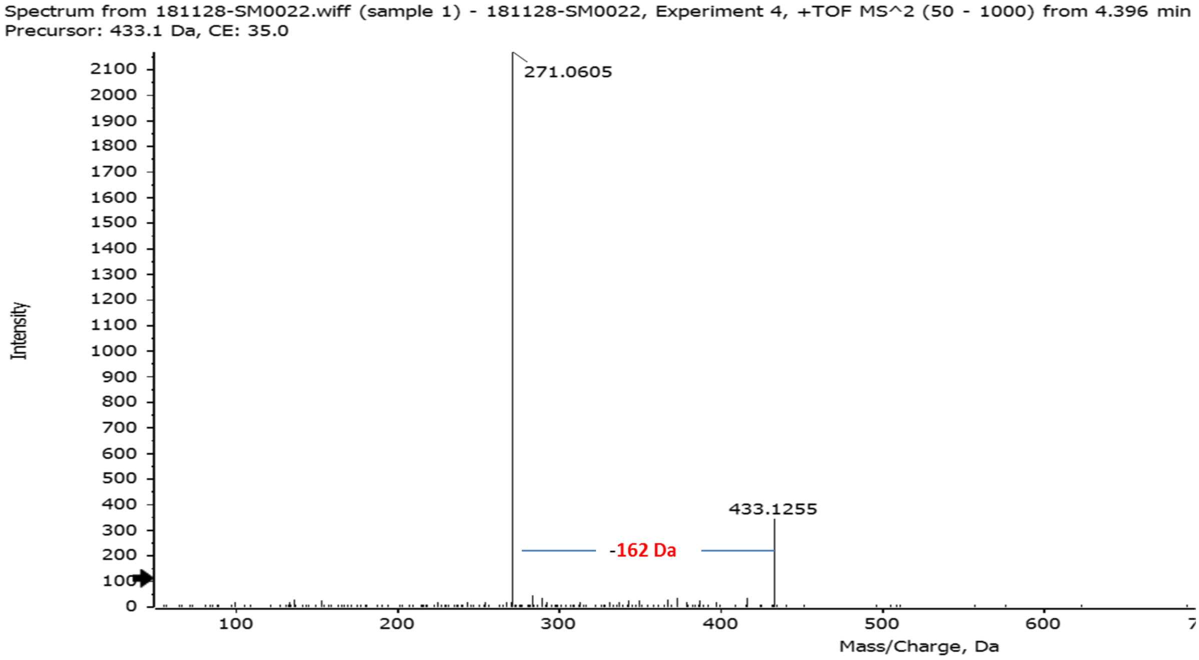

Figure S6. MS/MS spectrum of peak 26: Apigenin-O-hexoside.

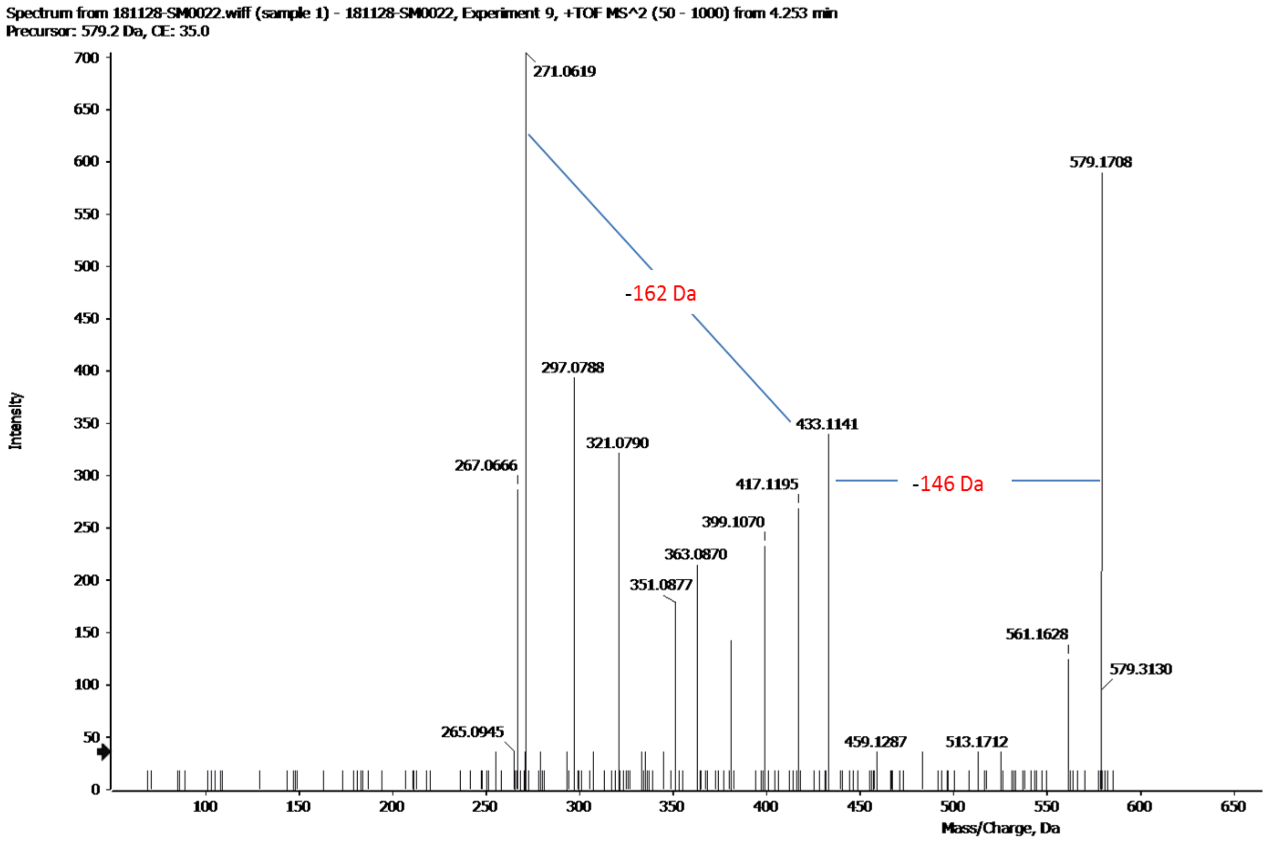

Figure S7. MS/MS spectrum of peak 25: Apigenin-O-hexoside-O-rhamnoside.

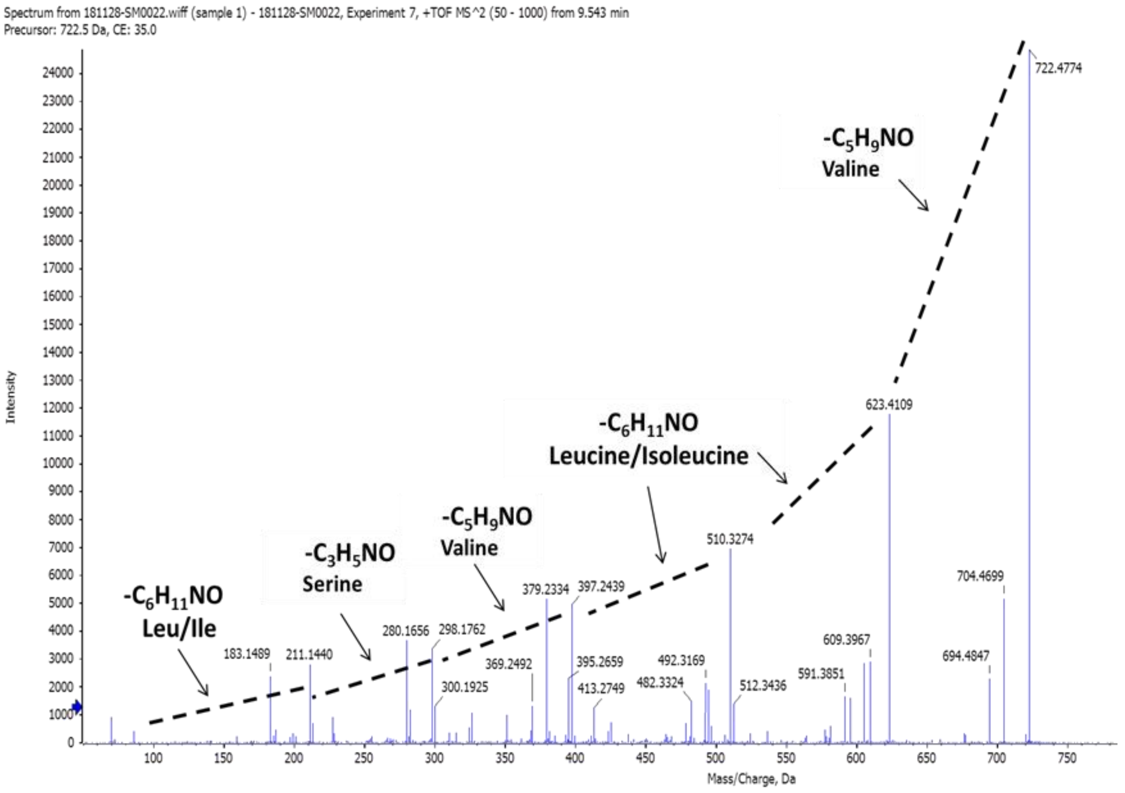

Figure S8. MS/MS spectrum of peak 43: A cycloheptapeptide.

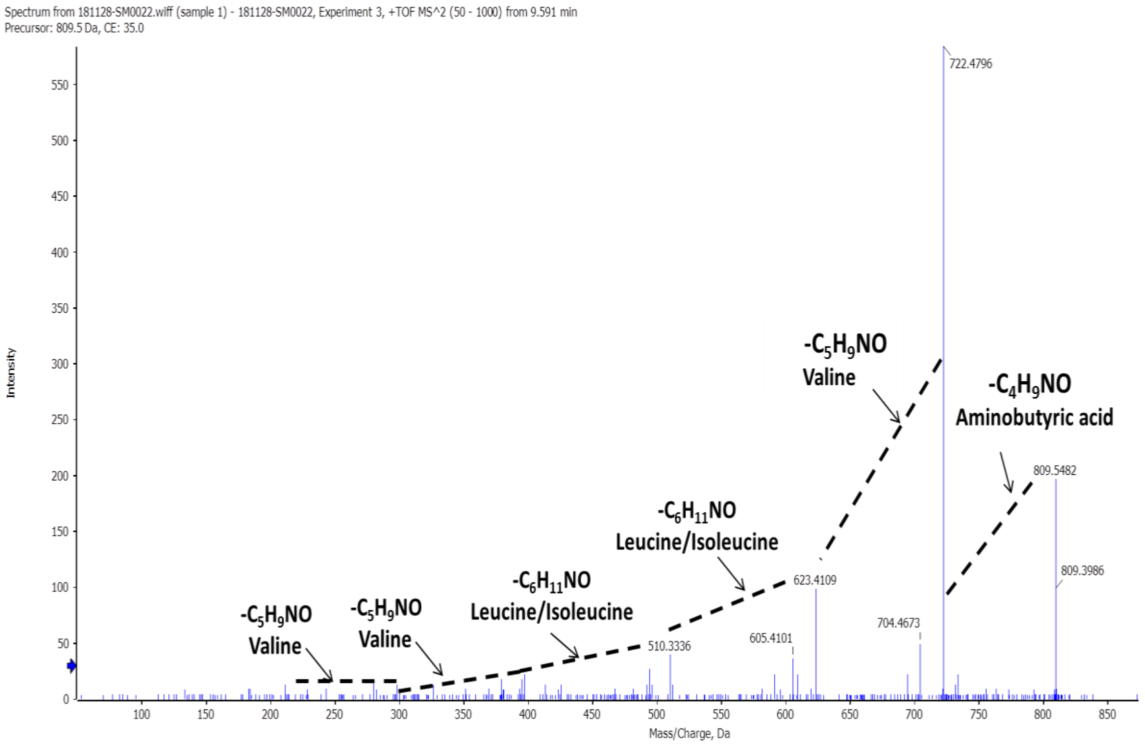

Figure S9. MS/MS spectrum of peak 45: A cyclooctapeptide.

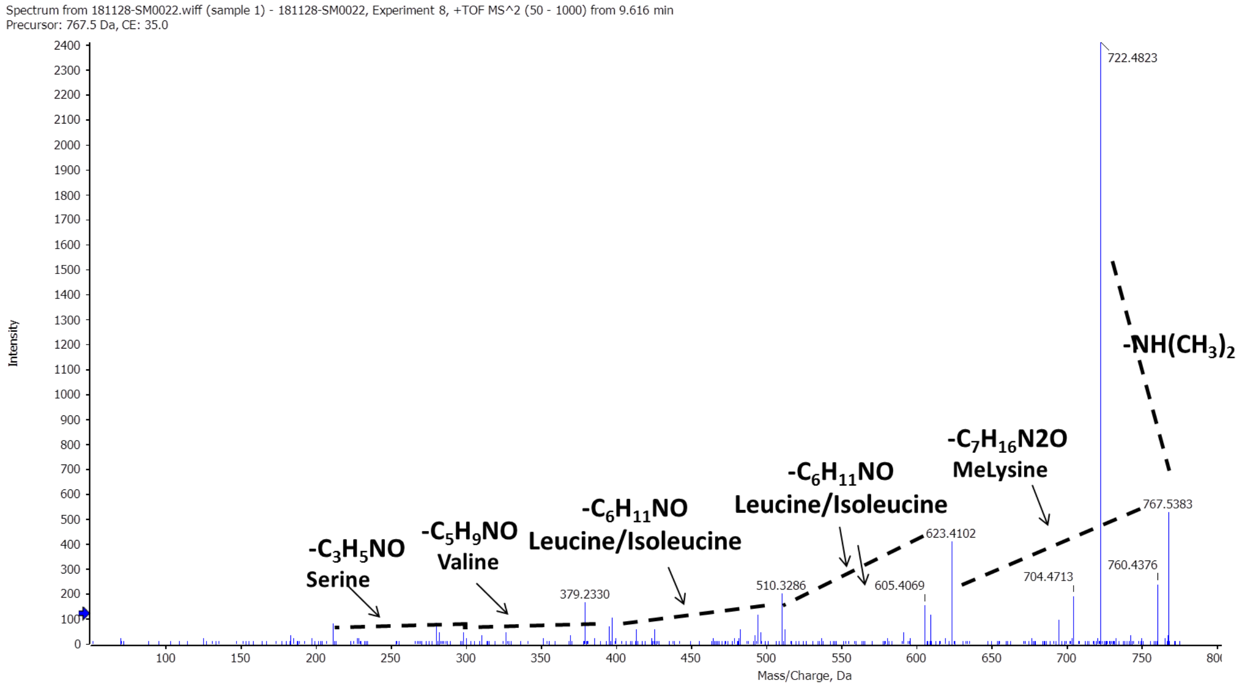

Figure S10. MS/MS spectrum of peak 46: A cycloheptapeptide.

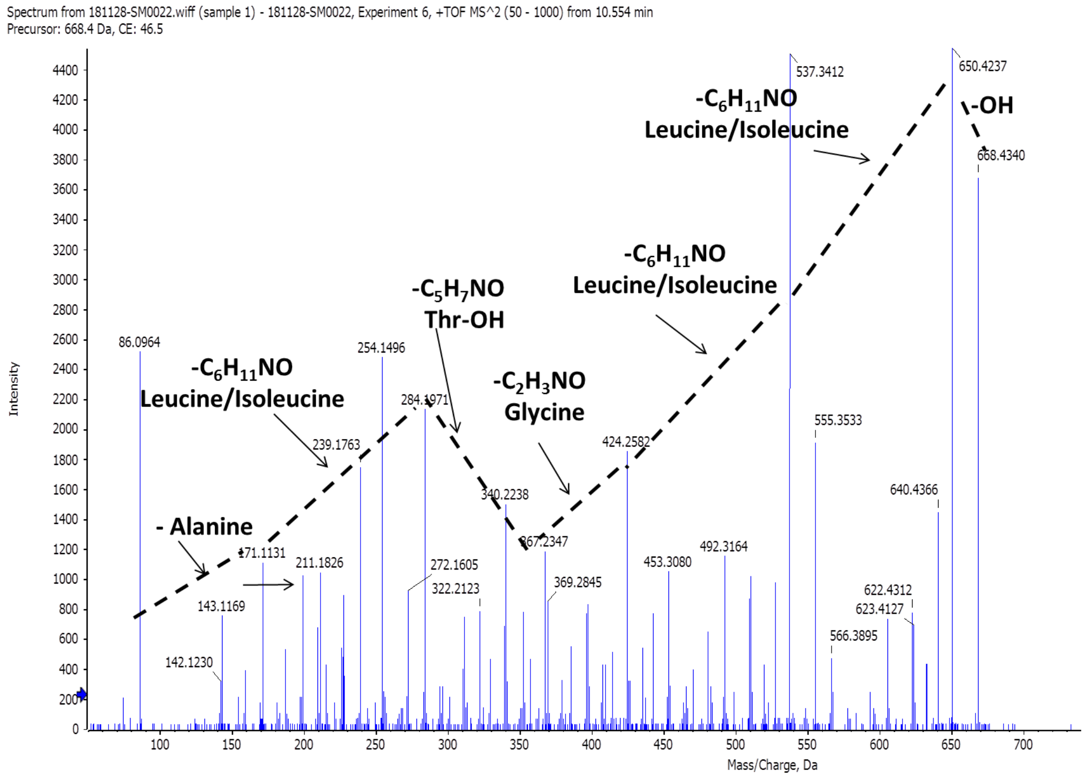

Figure S11. MS/MS spectrum of peak 46: A cycloheptapeptide.

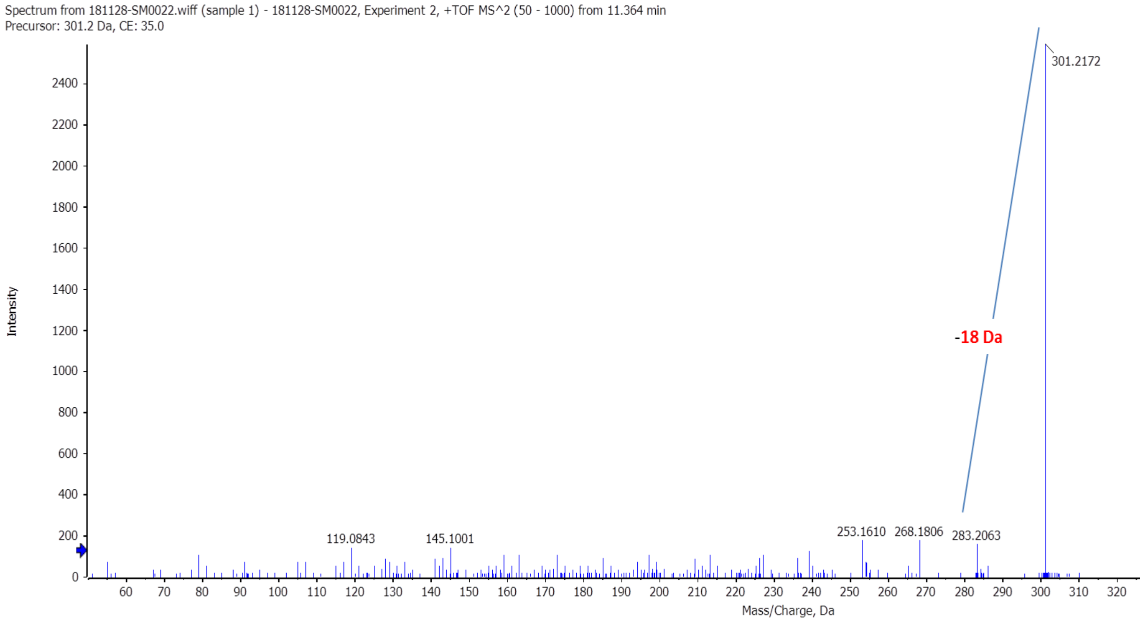

Figure S12. MS/MS spectrum of peak 59: Spruceanol.

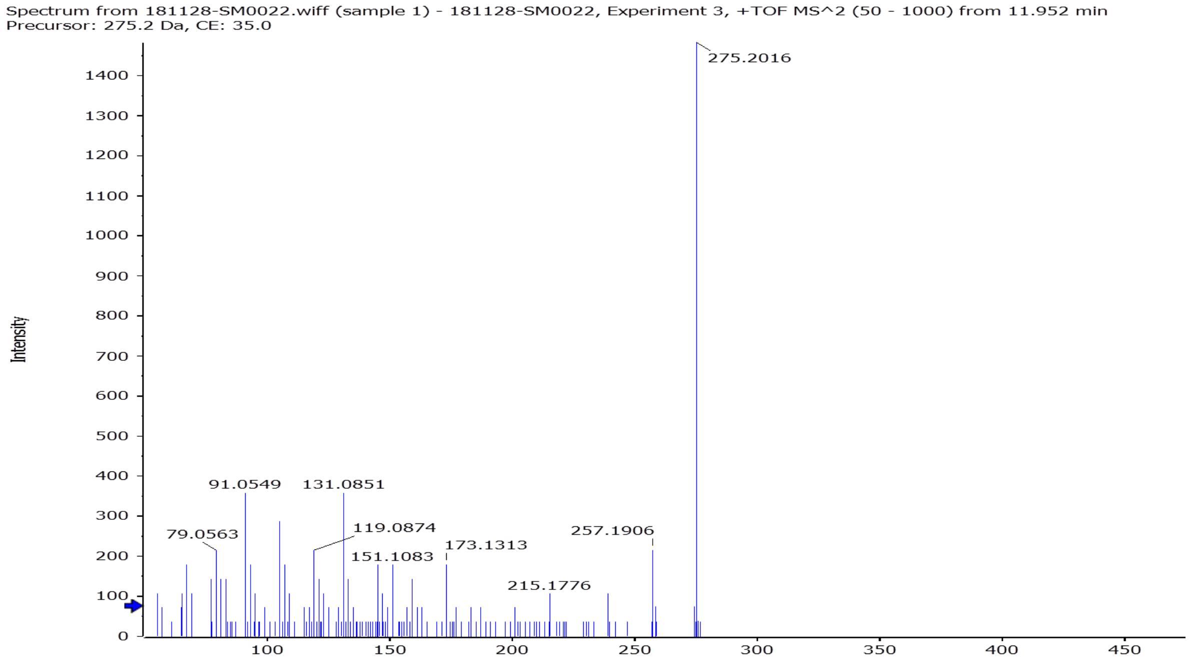

Figure S13. MS/MS spectrum of peak 61.

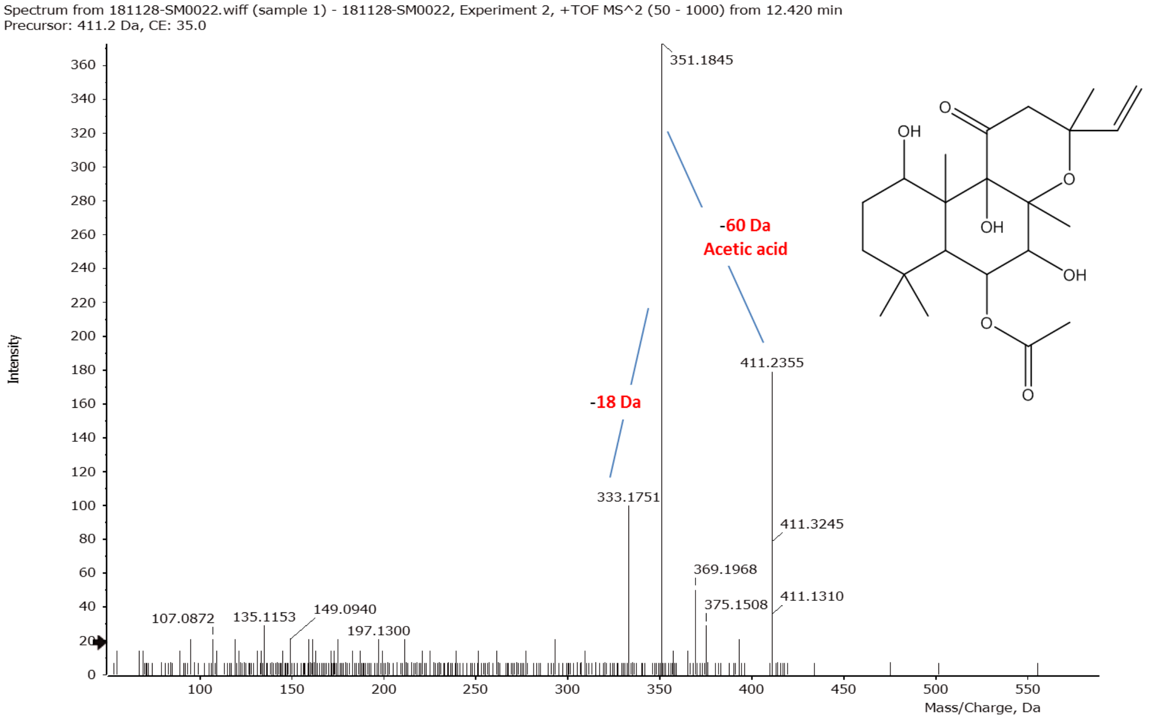

Figure S14. MS/MS for peak 67: Isoforskolin.

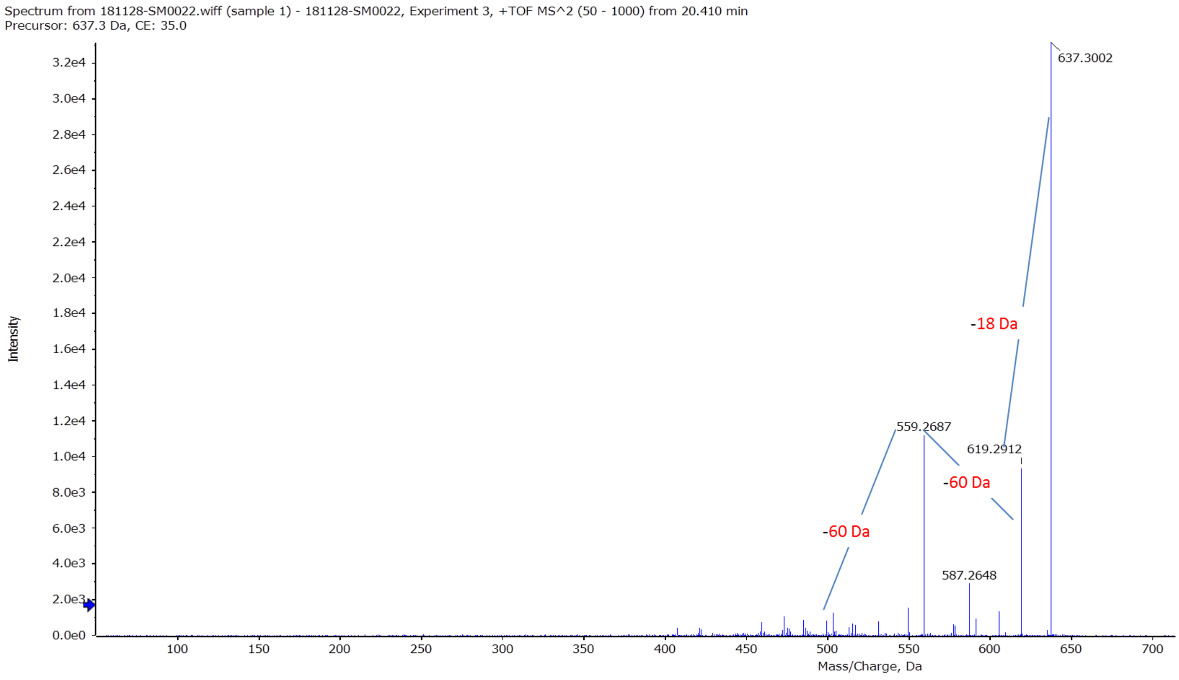

Figure S15. MS/MS for peak 114: Premyrsinol/pedittithin derivatives.

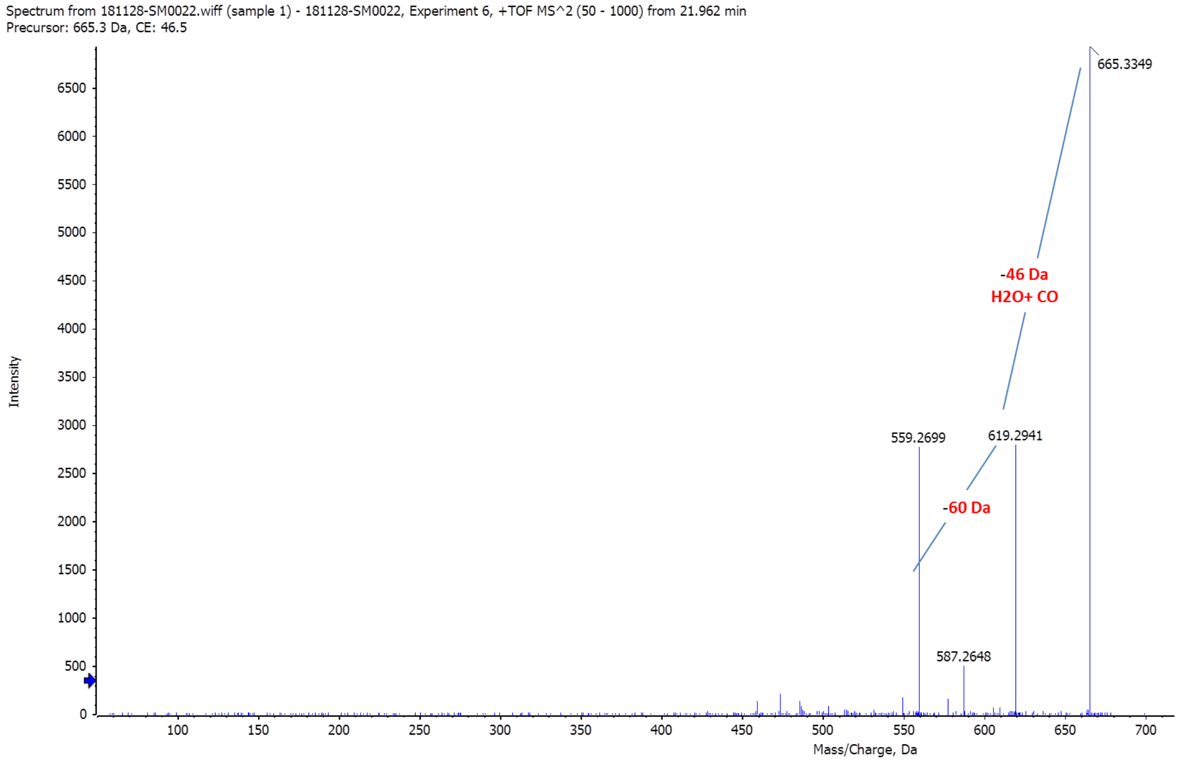

Figure S16. MS/MS of peak 125: Premyrsinol/pedititthin derivative.

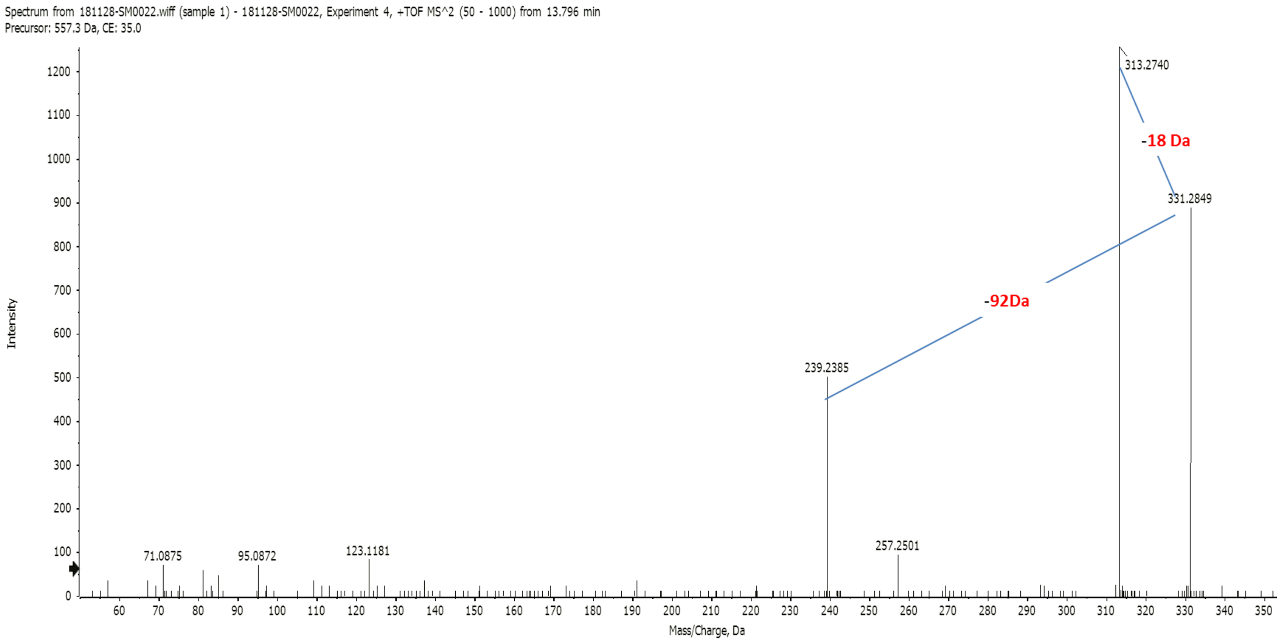

Figure S17. MS/MS of peak 82: Monopalmitin.

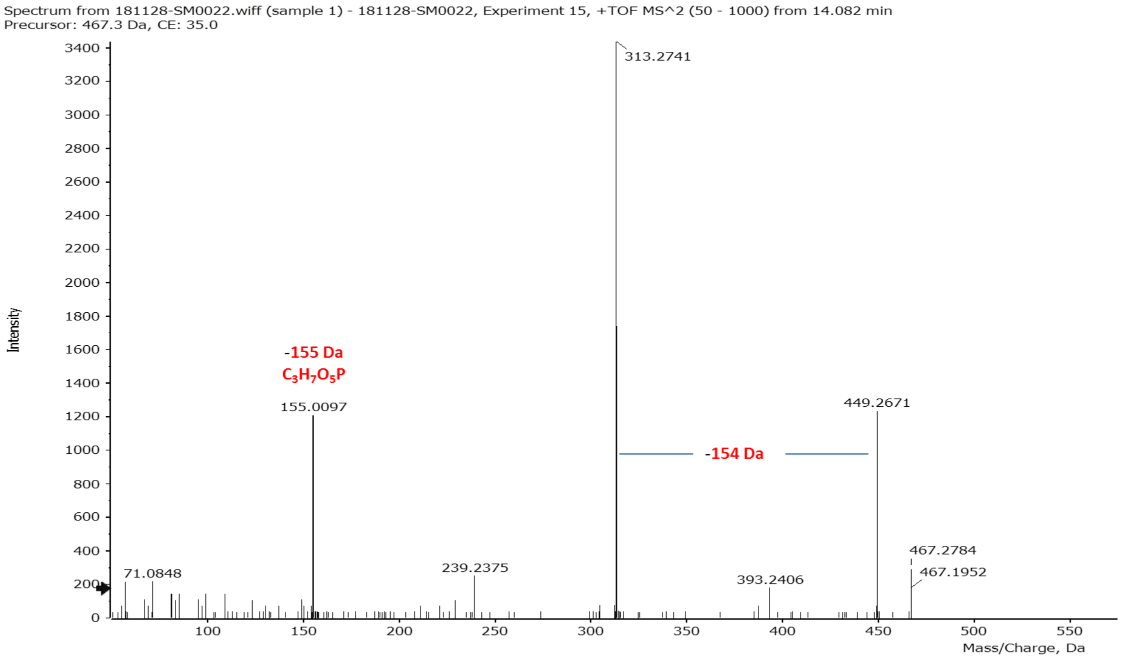

Figure S18. MS/MS of peak 84: Phosphatidic acid (10:0/9:0).

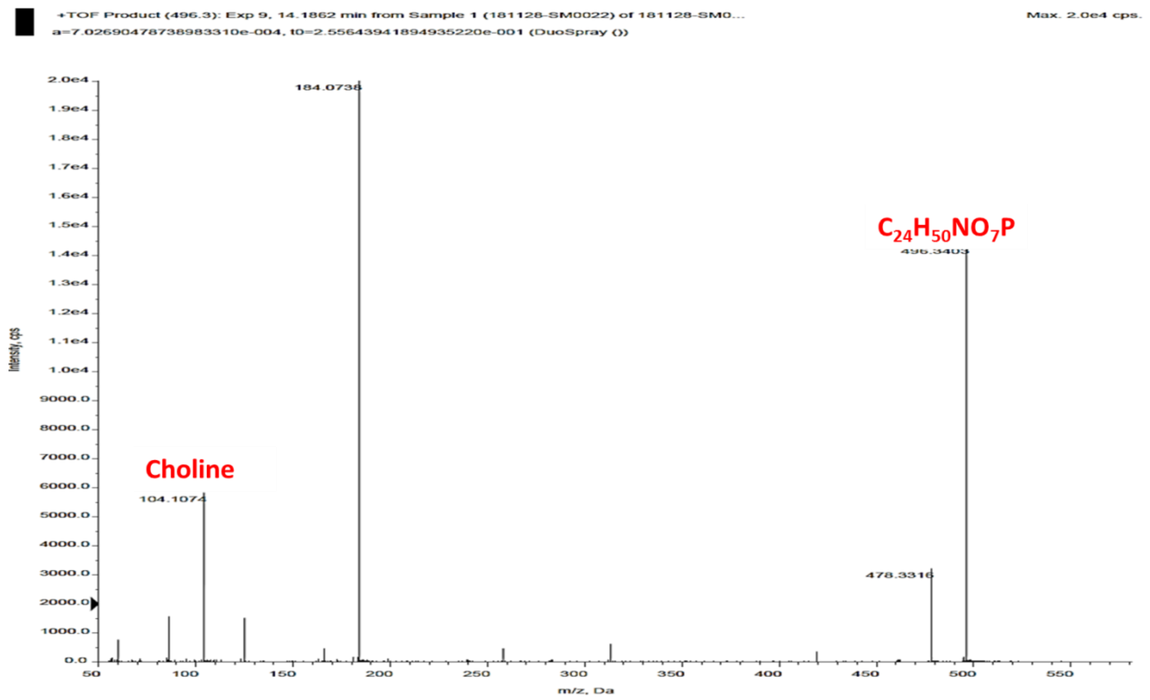

Figure S19. MS/MS of peak 86: Lysophosphatidylcholine (16:0/0:0).

Table S1. Analysis of high resolution MS/MS Q-TOF fragments for newly identified cyclic peptides.

Peak No.	*m/z*	Calculated formula	MS data *m/z*	calculated formula	Neutral loss	Corresponding amino acid
43/45	722.4774	$C_{36}H_{64}N_7O_8$	623.4151	$C_{31}H_{55}N_6O_7$	−99 Da	Val
			510.3274	$C_{25}H_{44}N_5O_6$	−113 Da	Leu/Ile
			397.2439	$C_{19}H_{33}N_4O_5$	−113 Da	Leu/Ile
			298.1762	$C_{14}H_{24}N_3O_4$	−99 Da	Val
			211.1440	$C_{11}H_{19}N_2O_2$	−87 Da	Ser
46	809.5482	$C_{40}H_{73}N_8O_9$	722.4796	$C_{36}H_{64}N_7O_8$	−87 Da	ABA
			623.4109	$C_{31}H_{55}N_6O_7$	−99 Da	Val
			510.3336	$C_{25}H_{44}N_5O_6$	−113 Da	Leu/Ile
			397.2439	$C_{19}H_{33}N_4O_5$	−113 Da	Leu/Ile
			298.1762	$C_{14}H_{24}N_3O_4$	−99 Da	Val
			211.1436	$C_{11}H_{19}N_2O_2$	−87 Da	Ser
47	767.5383	$C_{38}H_{71}N_8O_8$	623.4102	$C_{31}H_{55}N_6O_7$	−144 Da	MeLys
			510.3286	$C_{25}H_{44}N_5O_6$	−113 Da	Leu/Ile
			397.2445	$C_{19}H_{33}N_4O_5$	−113 Da	Leu/Ile
			298.1805	$C_{14}H_{24}N_3O_4$	−99 Da	Val
			211.1434	$C_{11}H_{19}N_2O_2$	−87 Da	Ser
55	668.4340	$C_{32}H_{58}N_7O_8$	555.3528	$C_{26}H_{47}N_6O_7$	−113 Da	Leu/Ile
			442.2628	$C_{20}H_{36}N_5O_6$	−113 Da	Leu/Ile
			385.2413	$C_{18}H_{33}N_4O_5$	−57 Da	Gly
			284.1949	$C_{14}H_{24}N_3O_4$	−101 Da	Thr
			171.1119	$C_8H_{15}N_2O_2$	−113 Da	Lec/Ile
			86.0964	$C_5H_{12}N$	−85 Da	Ala

ABA : amino butyric acid, Ala : alanine, Gly : glycine, Ile : isoleucine, Leu: leucine, Lys : lysine, Ser : serine, Thr : threonine, Val : valine. Isoleucine and Leucine cannot be differentiated based on their high resolution mass.
